# Event-Based Communication and Finite-Time Consensus Control of Mobile Sensor Networks for Environmental Monitoring

**DOI:** 10.3390/s18082547

**Published:** 2018-08-03

**Authors:** Yu Hu, Qiang Lu, Yanzhu Hu

**Affiliations:** 1 School of Automation, Beijing University of Posts and Telecommunications, Beijing 100876, China; hu_yu@bupt.edu.cn (Y.H.); yanzhu_hu@bupt.edu.cn (Y.H.); 2 School of Automation, Hangzhou Dianzi University, Hangzhou 310000, China

**Keywords:** finite-time stability, mobile sensor networks, consensus control, event-based communication

## Abstract

This paper deals with the problem of environmental monitoring by designing a cooperative control scheme for mobile sensor networks. The proposed cooperative control scheme includes three main modules: a wireless communication module, a direction decision module, and a motion control module. In the wireless communication module, an event-based communication rule is proposed, which means that mobile sensor nodes do not send their positions, velocities, and the data of environmental attributes to the other sensor nodes in real-time for the coordination and control of mobile sensor networks. Due to using the event-based communication rule, the communication bandwidth can be saved. In the direction decision module, a radial basis function network is used to model the monitored environment and is updated in terms of the sampled environmental data and the environmental data from the other sensor nodes by the wireless communication module. The updated environment model is used to guide the mobile sensor network to move towards the region of interest in order to efficiently model the distribution map of environmental attributes, such as temperature, salinity, and pH values for the monitored environment. In the motion control module, a finite-time consensus control approach is proposed to enable the sensor nodes to quickly change their movement directions in light of the gradient information from the environment model. As a result of using the event-based communication rule in the wireless communication module, the proposed control approach can also lower the updating times of the control signal. In particular, the proposed cooperative control scheme is still efficient under the directed wireless communication situation. Finally, the effectiveness of the proposed cooperative control scheme is illustrated for the problem of environmental monitoring.

## 1. Introduction

**Figure 1:**
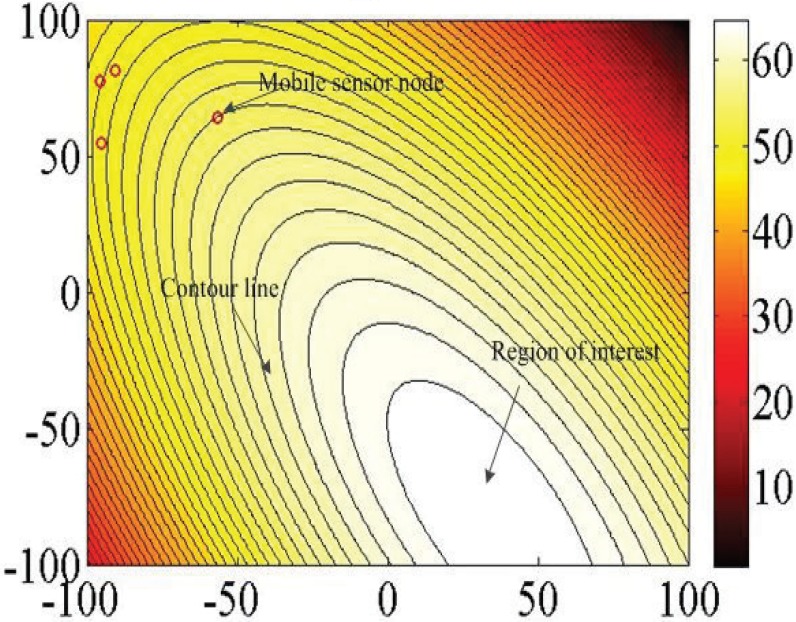
A simulated contour map of an environmental attribute.

In order to gain a better understanding of the environmental states including physical, chemical, and biological parameters, environmental monitoring has obtained much attention from scientists [1,2,3]. In particular, environmental monitoring involves a process of collecting data related to environmental attributes, such as temperature, salinity, pH values, odor concentration, and so on [1,3,4,5], and the goal is to build a data map to describe the environmental states. Figure 1 shows the contour map of environmental attributes where the red circle denotes the mobile sensor node and the surface color refers to concentration of an environmental attribute. The mobile sensor nodes need to communicate with each other by wireless networks in order to exchange the collected data on environmental attributes, positions, and velocities of sensor nodes such that sensor nodes hold the cooperative fashion and move toward the region of interest, i.e., the region with the maximum information while building the data map.

With technological advances in mobile sensor networks (such as autonomous surface vehicles), the investigation of how to control mobile sensor nodes to collect the data of environmental attributes and build environmental model, has received increasing attention from environmental scientists and control engineers [6,7,8,9,10,11,12]. In particular, compared with a single mobile sensor, multiple mobile sensors can collect multiple pieces of sampling information while increasing the sensing coverage, which can potentially improve the environmental monitoring performance [6,8,9,10,11,12]. Recently, there has been a growing interest in the coordination and control of multiple mobile sensors [6,7,9,10]. As a result, the control of a group of mobile sensors to monitor the situation of environment has also been studied [1,2,3]. However, there are a few challenges to address. The first challenge lies in the design of communication rule. Since real-time communication among sensors is not necessary, which means that some sensor states can be neglected while maintaining cooperative control performance, we wish to seek an event-based communication rule that saves the communication bandwidth. The second challenge is the design of the finite-time controller under the event-based communication rule. In particular, the designed finite-time controller is also required in the directed communication situation.

Therefore, we propose a cooperative control scheme including three main modules: a wireless communication module, a direction decision module, and a motion control module for the problem of environmental monitoring. Our objective is to construct and maintain an environmental model by controlling the mobile sensor network to collect data of environmental attributes with the greatest amount of information. This paper includes the following contributions: (1) We propose an event-based communication rule, that is, whether or not the states of sensors should be transmitted is determined by the states of its neighbors at the latest event time and the error between the current state and the latest transmitted state. Due to using the event-based communication rule, communication resources can be saved. (2) We propose a finite-time consensus control approach under the event-based communication rule which enables the mobile sensor nodes to quickly adjust the movement direction such that the sensor nodes can move towards the region with the greatest information while saving computational resources. (3) We give parameter conditions such that the proposed cooperative control scheme is still efficient under the directed wireless communication situation, which means that the networked control system of sensors with the proposed cooperative control scheme is stable.

The following is a brief introduction to the content of this paper. In Section 2, we describe the related work for event-triggered approaches and finite-time control approaches. In Section 3, we propose a cooperative control scheme and introduce three main modules. Then, we give an example to show the performance capabilities of the proposed cooperative control scheme. In Section 4, based on the proposed cooperative control scheme, we apply mobile sensor networks to deal with the problem of environment monitoring. The final remarks are given in Section 5.

## 2. Related Work

Consensus control approaches as a class of fundamental methods for mobile sensor networks have also been well studied [6,7,13,14]. In the face of some applications that require a fast convergence rate and high control accuracy [15,16,17], how to establish exponential convergence consensus approaches with finite settling times is necessary and has resulted in the appearance of finite-time consensus approaches. It is worth mentioning that finite-time stable systems show better performance capabilities with disturbance rejection [15,18,19]. As a result, finite-time consensus protocols have been proposed for mobile sensor networks [20,21,22]. For example, Cao et al. (2010) [20] used a control scheme with two levels—a finite-time estimating level and a finite-time tracking level—to hold the formation tracking of sensor nodes with single-integrator dynamics where the sliding mode approach is given to realize the finite-time control. Cortés et al. (2006) [21] proposed a finite-time convergent gradient control approach in order to control the network consensus in finite time where the states of sensors evolve based on single-integrator dynamics. Hui et al. (2008) [22] designed a finite-time consensus algorithm for nonlinear dynamical networks.

It should be noted that the above finite-time control approaches were developed for sensor nodes with single-integrator dynamics [20,21,22]. Then, these finite-time control approaches were further extended for sensor nodes with double-integrator dynamics [23]. For example, Hui et al. (2011) [23] designed a finite-time rendezvous algorithm using sign functions for nonlinear dynamical networks where sensor nodes evolve in terms of double-integrator dynamics. An important characteristic of the aforementioned finite-time control approaches is that they directly use sign functions to obtain discontinuous control inputs. However, chattering issues may be produced due to discontinuous control inputs [20,21,23] which leads to the appearance of continuous finite-time consensus control approaches [3,15,17,24,25,26,27]. For example, Wang and Hong (2008) [26] proposed several continuous finite-time consensus control approaches and gave the corresponding stability of double-integrator dynamics. Li et al. (2011) [15] developed a continuous finite-time consensus control approach by using a power function on the position and velocity of sensors. Lu et al. (2014) [3] designed a two-level control scheme where the function of the first level is to realize the finite-time cooperative control, and the function of the second level is to realize the finite-time tracking control. The designed control scheme is applied to the problem of odor source localization.

It should be pointed out that reducing the number of actuator updates may be preferable for some practical applications where some embedded processors with limited resources are employed [28,29,30,31,32,33,34]. Clearly, the above finite-time consensus control schemes need to continuously update control signals and cannot provide any support for this case. One possible method to tackle the issue is to use event-triggered consensus control schemes [35,36,37,38,39,40]. By adjudging an event triggering condition on state-dependent errors, whether or not control signals should be updated can be determined. For example, Dimarogonas et al. (2012) [35] designed an event-triggered consensus control approach and showed a smaller updating number while reaching consensus for the states of sensor nodes. Yi et al. (2017) [40] further developed a distributed event-triggered control approach for a directed communication situation. On the basis of the research results, references [40], Lu et al. (2017) [11] proposed an event-triggered finite-time consensus control approach for mobile sensor networks in order to deal with the problem of environmental monitoring.

However, the event-triggered consensus control schemes still need continuous-time communication between sensor nodes which may result in greater requirements for communication bandwidths. However, not all transmitted signals from neighboring nodes help to ensure the required control performance [10,41,42]. In order to release limited network resources to other communication tasks, some redundant signals can be avoided. Thus, several event-triggered communication schemes have also been proposed, where the states of sensors are broadcasted only when events are triggered [10,41,42]. Using this kind of event-triggered communication, schemes can not only reduce the updating times of control signals, but can also save network resources. For example, Zhu and Jiang (2015) [42] presented an event-based leader-follower consensus approach for sensor nodes with input time delay where data are transmitted only when the event occurs. Li et al. (2015) [10] developed an event-triggering sampling based consensus control approach for sensor nodes with second-order dynamics, where the communication bandwidth can be saved. A recent survey report shows more results about the event-triggered control schemes [43]. However, it is worth mentioning that the aforementioned consensus control approaches with event-triggered communication schemes cannot maintain the finite-time convergence of sensors’ states. Moreover, these consensus control approaches are also inefficient under directed communication situations, which means that the networked control system with the aforementioned consensus control approaches is not stable under directed communication situations.

## 3. Cooperative Control Scheme for Mobile Sensor Networks

### 3.1. Cooperative Control Scheme

**Figure 2:**
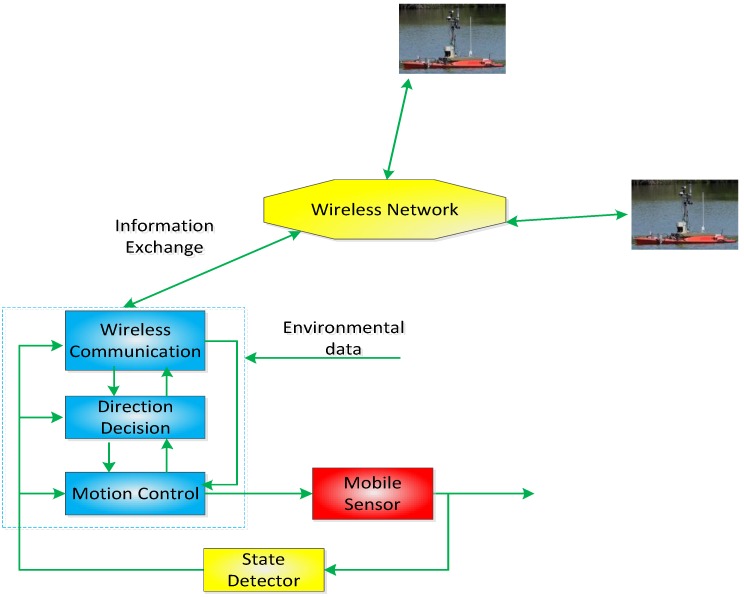
A cooperative control scheme.

In the following text, we propose a cooperative control scheme (shown in Figure 2), which includes three main modules: a wireless communication module, a direction decision module, and a motion control module.

From Figure 2, one can see that the mobile sensor nodes, such as autonomous surface vehicles, can exchange information with each other through wireless networks. Specifically, in the wireless communication module, the positions and velocities of sensor nodes and environmental data can be sent to other sensor nodes based on wireless communication. Correspondingly, these data can be received from other sensor nodes and be given to the direction decision module and motion control module. In the direction decision module, based on the environmental data and states of sensor nodes, sensor nodes can make a decision on their movement direction and transmit the corresponding decision data to the motion control module and the wireless communication module. In the motion control module, based on the direction decision of the sensor node, the sensor node is controlled to move towards the region with the greatest amount of information.

### 3.2. Wireless Communication Module and Event-Based Communication Rule

**Figure 3:**
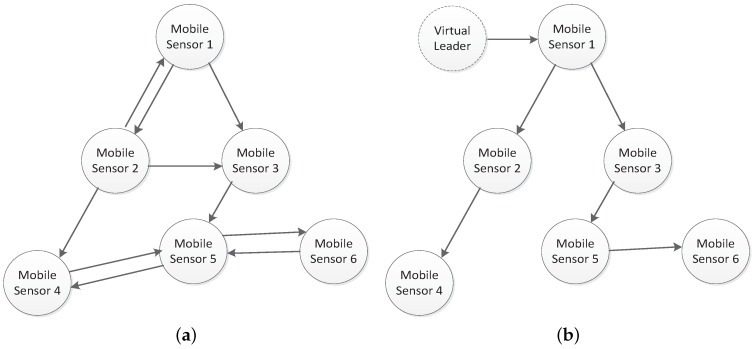
(**a**) A directed communication topology among six sensor nodes and (**b**) a directed spanning tree where the virtual leader is a root node.

Due to the possible failure and data loss in the transmitter and receiver of the wireless communication module of sensor nodes, a directed communication situation may exist among sensor nodes, which means that communication topologies among sensor nodes may be directed. From Figure 3a, one can see that, due to disturbance and time-delay, the data from the mobile sensor 3 are not received by the mobile sensor 1. Hence, a directed communication link exists between the mobile sensor 1 and mobile sensor 3. With the similar reason, directed communication link occurs between mobile sensor 2 and mobile sensor 3.

In order to model the directed communication topology, we used the directed graph $G_{n}\left(W,E,P\right)$, where $W=\{w_{1},w_{2},\cdots ,w_{n}\}$ denotes a set formed by mobile sensor nodes; E⊆W×W represents the set of communication links; $P=[p_{ij}]$ is a weighted adjacency matrix; and $p_{ij}\geq 0$ describes the communication quality. For the adjacency matrix, *P*, if the ith sensor node obtains the information from the jth sensor node, then $p_{ij}>0$; otherwise, $p_{ij}=0$. Figure 3b, shows a virtual leader that communicates with the other sensor nodes and can be put in any sensor node. The virtual leader does not exist in real applications, and is introduced to help sensor nodes reach consensus, and one can also control the final convergence velocity of mobile sensor network so as to obtain a leader-follower formation. For the directed graph, if the *i*th node can transmit the data to the *j*th node, the *i*th node is called the parent node and the *j*th node is called the child node. In addition, the root node has no parent and has a directed path to every other node. In Figure 3b, the virtual leader is a root node since directed paths to other nodes exist. A directed path is a directed graph in which every node has one parent node except for the root node. For example, the four nodes, including the virtual leader, mobile sensor 1, mobile sensor 2, and mobile sensor 4 form a directed path where the virtual leader is a root node.

A directed tree has the following characteristics: (i) the root node has no parent node; (ii) each node has only one parent node; and (iii) the root node has a directed path to every other node. Let the directed spanning tree $G^{s}\left(W^{s},E^{s},P^{s}\right)$ be a subgraph of $G_{n}\left(W,E,P\right)$ such that $G^{s}\left(W^{s},E^{s},P^{s}\right)$ is a directed tree and $W^{s}=W$. Moreover, $G_{n+1}=G_{n}\cup \left\{w_{0}\right\}$ is an expansion of $G_{n}\left(W,E,P\right)$, where $w_{0}$ represents the virtual leader. If the virtual leader can communicate with the ith sensor node, $p_{i0}>0$; otherwise, $p_{i0}=0$ (i=1,…,n). $L_{G_{n}}=\left[s_{ij}\right]\in R^{n\times n}$ is a Laplacian matrix, which can be calculated by

(1) $$\begin{array}{c} s_{ij}=\left\{\begin{array}{cc} \sum_{j=1,j\neq i}^{N}p_{ij}, & i=j\\ -p_{ij}, & i\neq j. \end{array}\right. \end{array}$$

The event-based time sequence $t_{0}^{i},t_{1}^{i},\cdots ,t_{s}^{i},\cdots$ (*s* is an index number, and *i* is the index number of the *i*th mobile sensor node. ) defined iteratively by

(2) $$\begin{array}{c} t_{s+1}^{i}=\inf\left\{t|t>t_{s}^{i},g_{i}\left(t\right)>0\right\} \end{array}$$

with

$$\begin{array}{cc} g_{i}\left(t\right)= & ∥M∥|\beta e_{ix}\left(t\right)+\gamma e_{iv}\left(t\right)|+p_{i0}\left|\beta e_{i0x}\left(t\right)+\gamma e_{i0v}\left(t\right)\right|\\ & -h(|\beta y_{i}\left(t_{s}^{i}\right)|+|\gamma z_{i}\left(t_{s}^{i}\right)|), \end{array}$$

where inf is the greatest lower bound; $g_{i}\left(t\right)$ is a function; β>0, h>0, γ>0, $e_{ix}\left(t\right)=x_{i}\left(t_{s}^{i}\right)-x_{i}\left(t\right)$, $e_{iv}\left(t\right)=v_{i}\left(t_{s}^{i}\right)-v_{i}\left(t\right)$, $e_{i0x}\left(t\right)=x_{0}\left(t_{s}^{i}\right)-x_{0}\left(t\right)$, $e_{i0v}\left(t\right)=v_{0}\left(t_{s}^{i}\right)-v_{0}\left(t\right)$, $y_{i}\left(t_{s}^{i}\right)=\sum_{j=0}^{n}p_{ij}\left(x_{j}\left(t_{s^{\prime }\left(t\right)}^{j}\right)-x_{i}\left(t_{s}^{i}\right)\right)$, and $z_{i}\left(t_{s}^{i}\right)=\sum_{j=0}^{n}p_{ij}\left(v_{j}\left(t_{s^{\prime }\left(t\right)}^{j}\right)-v_{i}\left(t_{s}^{i}\right)\right)$, $s^{\prime }\left(t\right)=\arg\min_{l\in Z_{0}^{+}t\geq t_{l}^{j}}\left\{t-t_{l}^{j}\right\}$ denotes time with the newest data from the *j*th sensor node; $x_{i}\left(t\right)$ and $x_{i}\left(t_{s}^{i}\right)$ are the positions of the *i*th sensor node at time *t* and triggering time $t_{s}^{i}$, respectively; $v_{i}\left(t\right)$ and $v_{i}\left(t_{s}^{i}\right)$ are the velocities of the *i*th sensor node at time *t* and triggering time $t_{s}^{i}$, respectively; $x_{0}\left(t\right)$ and $x_{0}\left(t_{s}^{i}\right)$ are the positions of the virtual leader at time *t* and triggering time $t_{s}^{i}$, respectively; $v_{0}\left(t\right)$ and $v_{0}\left(t_{s}^{i}\right)$ are the velocities of the virtual leader at time *t* and triggering time $t_{s}^{i}$, respectively; $p_{ij}$ is the communication weight between the *i*th sensor node and the *j*th sensor node; $v_{j}\left(t_{s^{\prime }\left(t\right)}^{j}\right)$ and $x_{j}\left(t_{s^{\prime }\left(t\right)}^{j}\right)$ are the velocity and position of the *j*th sensor node at the latest triggering time $t_{s^{\prime }\left(t\right)}^{j}$, respectively; $Z_{0}^{+}$ is a positive integer set; $t_{1}^{j},t_{2}^{j},\ldots ,t_{l}^{j}$ is a time sequence for the *j*th sensor node; ∥·∥ denotes 2-norms and |·| refers to the absolute value symbol. Note that $t_{s^{\prime }\left(t\right)}^{0}=t_{s}^{i}$ and at least one sensor node that can communicate with the virtual leader exists. $M=L_{G_{n}}+\mathrm{diag}\left\{p_{10},\ldots ,p_{n0}\right\}$. $L_{G_{n}}$ denotes the non-symmetrical Laplacian matrix of directed graph $G_{n}\left(W,E,P\right)$, and $p_{i0}$ represents the communication link between the ith (i=1,…,n) sensor node and the virtual leader. In addition, the proposed event-based rule (2) can reduce communication burdens between sensor nodes since the information of the jth (j=1,…,n, j≠i) sensor node is required only at $t=t_{s^{\prime }\left(t\right)}^{j}$.

From (2), one can see that when $∥M∥|\beta e_{ix}\left(t\right)+\gamma e_{iv}\left(t\right)|+p_{i0}|\beta e_{i0x}\left(t\right)+\gamma e_{i0v}\left(t\right)|>h(|\beta y_{i}\left(t_{s}^{i}\right)|+|\gamma z_{i}\left(t_{s}^{i}\right)\left|\right)$, the event-triggered condition is satisfied. Correspondingly, the new state of the ith sensor node is broadcasted into its neighbors based on wireless communication networks. Moreover, one can also see that when the event-triggered condition is not satisfied, the item $h(|\beta y_{i}\left(t_{s}^{i}\right)|+|\gamma z_{i}\left(t_{s}^{i}\right)|)$ stays constant which means that the ith sensor node does not need to send its state into its neighbors. It should be pointed out that the use of the event-based communication rule means that the mobile sensor nodes are not required to continually send data to communication networks such that the energy consumption of sensor nodes can be decreased [44,45,46].

**Remark** **1.** *Compared with the event-based communication rules in [10,42], proposed event-based communication rule (2) has the following characteristics: (i) The expression form of proposed event-based communication rule (2) is simple and concise. (ii) The parameters from proposed event-based communication rule (2) can be easily set while the parameters from references [10,42] are hard to calculate. (iii) Under proposed event-based communication rule (2), we can design a finite-time consensus controller that is also efficient for directed communication situations. For the event-based communication rules in references [10,42], the given consensus controller cannot enable the states of sensor nodes to reach consensus in finite time.*

**Remark** **2.** *Note that event-based condition (2) is estimated in real-time. When the event-triggered condition is satisfied, the new state of the ith sensor node is broadcasted into its neighbors based on wireless communication networks. The event-triggered time is recorded and put into the event-triggered time sequence $t_{0}^{i},t_{1}^{i},\cdots ,t_{s}^{i},\cdots$.*

### 3.3. Direction Decision Module and Environmental Model

In order to model the monitored environment and obtain the data map of environmental attributes, a radial basis function network [11] is employed as

(3) $$\begin{array}{ccc} q_{i}\left(x\right) & = & \sum_{j=1}^{m}\theta_{j}\exp\left(\frac{-∥x-\omega_{j}{∥}^{2}}{2\sigma_{j}^{2}}\right) \end{array}$$

where *m* is the number of radial basis functions; $\theta_{j}$ is the weight of the jth radial basis function; $q_{i}\left(x\right)$ is the estimated value based on the radial basis function network; *x* is the position of sensor nodes; $\omega_{j}$ is the center of the jth radial basis function; and $\sigma_{j}$ is the width of the jth radial basis function.

The latest data obtained from the sensor node is used to update the weights of the radial basis function network shown in (4).

(4) $$\begin{array}{c} \min_{\theta_{j},j=1,\ldots ,m}\;\sum_{l=1}^{n}p_{il}{\left|r_{l}\left(x_{l}\right)-q_{i}\left(x_{l}\right)\right|}^{2} \end{array}$$

where $r_{l}\left(x_{l}\right)$ is the actual value obtained from the lth sensor node at position $x_{l}$, and $q_{i}\left(x_{l}\right)$ is the estimated value calculated from the environmental model (3). If the ith sensor node cannot get the information sent by the lth sensor node, then $p_{il}=0$; otherwise, $p_{il}=1$. In terms of the environmental model (3), the reference velocity is given by

(5) $$v_{ir}=\lambda \frac{\partial q_{i}\left(x\right)}{\partial x}{|}_{x=x_{i}}$$

where λ=20 in the following applications. The reference velocity, $v_{ir}$, can guide the *i*th sensor node to move towards the region with the greatest amount of information.

### 3.4. Motion Control Module and Finite-Time Control

A mobile sensor network is composed of *n* identical sensor nodes. The dynamics of the sensor node are described as

(6) $$\begin{array}{c} \left\{\begin{array}{ccc} {\dot{x}}_{i}\left(t\right) & = & v_{i}\left(t\right)\\ {\dot{v}}_{i}\left(t\right) & = & u_{i}\left(t\right) \end{array}\right. \end{array}$$

where $x_{i}\left(t\right)$ and $v_{i}\left(t\right)$ i=1,2,…,n are the position and velocity of the ith sensor node, respectively; $u_{i}\left(t\right)$ is the corresponding control signal; and *n* is the number of the sensor node. $x_{0}\left(t\right)$ represents the virtual leader’s position and $v_{0}\left(t\right)$ represents the virtual leader’s velocity. The kinematics of the virtual leader are obtained by

(7) $$\begin{array}{c} {\dot{x}}_{0}\left(t\right)=v_{0} \end{array}$$

where $v_{0}$ is a constant. The corresponding finite-time consensus controller is

(8) $$\begin{array}{cc} u_{i}\left(t\right)= & \mathrm{sig}{\left(\sum_{j=0}^{n}p_{ij}(\beta \left(x_{j}\left(t_{s^{\prime }\left(t\right)}^{j}\right)-x_{i}\left(t_{s}^{i}\right)+\gamma \left(v_{j}\left(t_{s^{\prime }\left(t\right)}^{j}\right)-v_{i}\left(t_{s}^{i}\right)\right)\right)\right)}^{\alpha }\\ & +\sum_{j=0}^{n}p_{ij}\left(\beta \left(x_{j}\left(t_{s^{\prime }\left(t\right)}^{j}\right)-x_{i}\left(t_{s}^{i}\right)\right)+\gamma \left(v_{j}\left(t_{s^{\prime }\left(t\right)}^{j}\right)-v_{i}\left(t_{s}^{i}\right)\right)\right) \end{array}$$

where $\mathrm{sig}{\left(c\right)}^{\alpha }=\mathrm{sign}\left(c\right){\left|c\right|}^{\alpha }$, sign(·) is a sign function, and 0<α<1.

Now, the following theorem is set up for the mobile sensor network which can show the parameters’ conditions such that the states’ consensus of mobile sensor nodes can be obtained.

**Theorem** **1.** *Consider mobile sensor network (6) and virtual leader (7) with the finite-time consensus protocol (8) and the event-triggered communication rule (2). Let $\mu_{\min}$ represent the minimum eigenvalue of $M+M^{T}$ where $k=\min_{i=1,\ldots ,n}\left\{\frac{\alpha }{\alpha +1}\left(\sum_{j=1}^{n}p_{ij}-\sum_{j=1}^{n}p_{ji}\right)+p_{i0},\frac{1}{2}\left(\sum_{j=1}^{n}p_{ij}-\sum_{j=1}^{n}p_{ji}\right)+p_{i0}\right\}$ and $M=L_{G_{n}}+\mathrm{diag}\left\{p_{10},\ldots ,p_{n0}\right\}$. If $G_{n+1}$ has a directed spanning tree with the virtual leader as the root, k is a positive constant, $\gamma >\sqrt{\frac{\beta }{\mu_{\min}}}$, $h<\min\{\frac{k\sqrt{k_{1}}}{∥M∥n^{\frac{1-\alpha }{2}}},\sqrt{\frac{k_{1}}{2}}\}$, and $\delta <\frac{\gamma^{2}\mu_{\min}-\beta }{2\gamma^{2}}$ where $\delta =∥M∥\sqrt{\frac{2h^{2}}{k_{1}-2h^{2}}}$. Then, the consensus controller (8) and the event-based communication rule (2) guarantees that $x_{i}\left(t\right)\to x_{0}\left(t\right)$ and $v_{i}\left(t\right)\to v_{0}$, ∀i∈{1,…,n} in finite time. The settling time is upper bounded by $\frac{2V{\left(0\right)}^{\frac{1-\alpha }{2}}}{k_{2}\left(1-\alpha \right)}$ where V(0) and $k_{2}$ can be calculated. Moreover, Zeno-behaviors are avoided before reaching consensus.*

**Proof.**  The proof of Theorem 1 can be found in Appendix D. ☐

It is worth mentioning that one can compute the upper bound of the convergence time according to Theorem 1, from which the initial states of mobile sensor networks have important impacts on the convergence time [3,15,17,26,27]. Moreover, the parameter α also affects the setting time and should be carefully chosen in (0,1). In addition, as a result of constructing the Lyapunov function, the obtained upper bound of the convergence time may be conservative.

Notice that the velocity ($v_{0}$) of the virtual leader shows the movement direction of the mobile sensor network. By setting the velocity ($v_{0}$) of the virtual leader, the velocities of sensor nodes can reach consensus in finite time through the directed communication structure. On the other hand, the movement direction of the virtual leader is required to guide the mobile sensor network to move towards the region with the greatest information. Hence, the velocity ($v_{0}$) of the virtual leader is set based on the reference velocity. If the virtual leader is put in the *i*th sensor node, then

(9) $$\begin{array}{c} v_{0}=v_{ir} \end{array}$$

where $v_{ir}$ is the reference velocity of the *i*th sensor node. In addition, in order to obtain a reasonable formation, we improved the proposed finite-time consensus control approach (10), as follows:

(10) $$\begin{array}{ccc} u_{i} & = & \mathrm{sig}{\left(\sum_{j=0}^{n}a_{ij}\left(\beta \left(\left(x_{j}\left(t_{s^{\prime }\left(t\right)}^{j}\right)-d_{j}\right)-\left(x_{i}\left(t_{s}^{i}\right)-d_{i}\right)\right)+\gamma \left(v_{j}\left(t_{s^{\prime }\left(t\right)}^{j}\right)-v_{i}\left(t_{s}^{i}\right)\right)\right)\right)}^{\alpha }\\ & & +\sum_{j=0}^{n}a_{ij}\left(\gamma \left(v_{j}\left(t_{s^{\prime }\left(t\right)}^{j}\right)-v_{i}\left(t_{s}^{i}\right)\right)+\beta \left(x_{j}\left(t_{s^{\prime }\left(t\right)}^{j}\right)-x_{i}\left(t_{s}^{i}\right)\right)\right) \end{array}$$

where $d_{i}$ is a random vector in which i=1,2,…,n, and $d_{0}=0$. Theorem 1 can guarantee that the proposed finite-time consensus control approach (10) with event-based communication rule (2) can enable $x_{i}\left(t\right)-d_{i}\to x_{0}\left(t\right)$, $x_{i}\left(t\right)-d_{i}\to x_{j}\left(t\right)-d_{j}$, and $v_{i}\left(t\right)\to v_{0}$, ∀i∈{1,…,n}. $x_{i}\left(t\right)-d_{i}\to x_{j}\left(t\right)-d_{j}$ can hold the reasonable formation of sensor nodes. $v_{i}\left(t\right)\to v_{0}$ can guide the mobile sensor network to track the velocity ($v_{0}$) of the virtual leader, and the velocity ($v_{0}$) is determined by (5) and (9).

**Remark** **3.** *Due to the use of the event-based communication rule, when the event-triggered condition is not satisfied, the control input in (10) is not calculated and keeps the last time input which implies that the number of actuator updates is reduced and the energy consumption of the sensor nodes is saved [47,48]. If the event-triggered condition is satisfied, the new control input in (10) needs to be calculated. Since the dynamics of sensor nodes are continuous-time double-integrator dynamics, we used a continuous-time event detector in theory which can be found in the proof process of Theorem 1 in Appendix D. However, in the following simulation and applications, we use a sampling time of 0.01 s to program the proposed cooperative scheme [49].*

The proposed cooperative control scheme is presented with the following Algorithm 1, and then we use an example to show the effectiveness of Algorithm 1.

**Algorithm 1** Cooperative Control Scheme /*Initialization*/ Initialize parameters *h*, β and γ, ∥M∥ of the event-based rule (2). Initialize the velocity ($v_{0}$) of the virtual leader and the adjusting parameter (λ). Initialize the parameters α and *n* for the finite-time consensus control in (8). /*Main Body*/ **repeat**  Detect the newest information from wireless communication networks.  Sample the environmental information and the states’ information.  Compute the event-triggered rule in (2).  **if**
$g_{i}\left(t\right)>0$
**then**   Send the environmental information and states’ information to their neighbors.   Update the control input in (8).  **end if**  **if**
$g_{i}\left(t\right)\leq 0$
**then**   Hold the lasted control input;  **end if**  Apply the control input to mobile sensor nodes. **until** The termination condition is satisfied.

**Figure 4:**
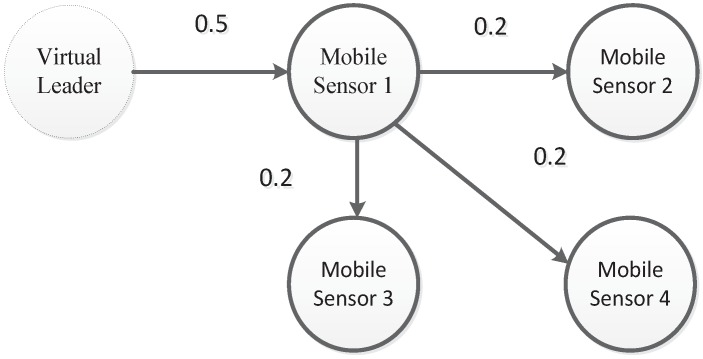
The directed communication topology with communication weights.

**Example** **1.** *Figure 4 shows a fixed communication topology ($G_{1}$) for four sensor nodes. We can clearly see that a directed spanning tree exists and the virtual leader (L) is the root node. The corresponding weights have also been labeled in Figure 4. For the communication topology ($G_{1}$), the eigenvalues of the matrix ($M+M^{T}$) are $\mu_{1}=0.2417$, $\mu_{2}=0.4$, $\mu_{3}=0.4$, and $\mu_{4}=1.1583$. Based on Theorem 1, the parameters of the proposed consensus controller are ∥M∥=0.6196, α=0.9, β=16.8, γ=22.6, k=0.2, $k_{1}=1$, and h=0.1. The initial positions of the sensor node are $x_{0}\left(0\right)=2$, $x_{1}\left(0\right)=-1.1$, $x_{2}\left(0\right)=1$, $x_{3}\left(0\right)=2.7$, and $x_{4}\left(0\right)=-0.9$. The initial velocities of the sensor node are $v_{0}\left(0\right)=0.8$, $v_{1}\left(0\right)=1.4$, $v_{2}\left(0\right)=0.5$, $v_{3}\left(0\right)=-1.2$, and $v_{4}\left(0\right)=-0.9$. Hence, the total run time is 7 s and its sampling time is 0.01 s which implies 700 iterations. Let $l_{i1}=h(|\beta y_{i}\left(t_{s}^{i}\right)|+|\gamma z_{i}\left(t_{s}^{i}\right)\left|\right)$ and $l_{i2}=∥M∥|\beta e_{ix}+\gamma e_{iv}|+a_{i0}\left|\beta e_{i0x}+\gamma e_{i0v}\right|$, i=1,…,4.*

**Figure 5:**
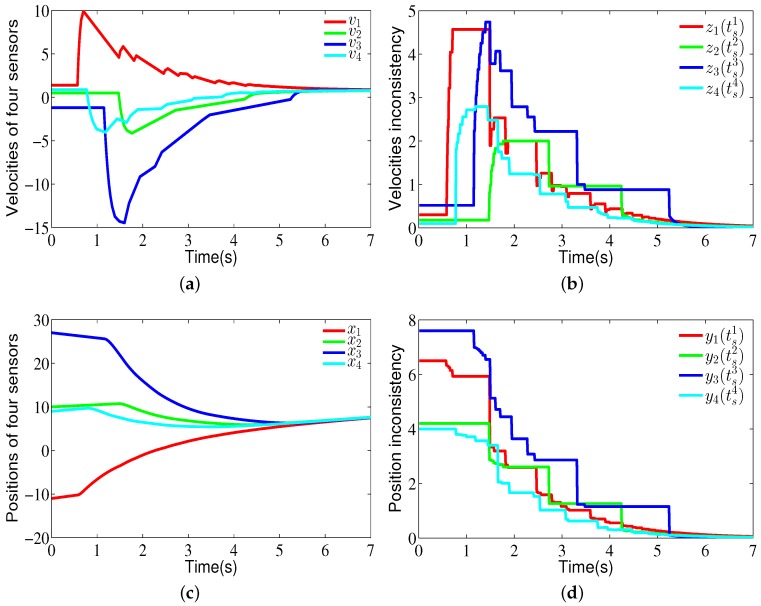
(**a**) shows the velocity state curves of the four sensor nodes and (**b**) shows the state curves of velocity inconsistency for the four sensor nodes. (**c**) shows the position state curves of the four sensor nodes, and (**d**) shows the state curves of position inconsistency for the four sensor nodes.

**Figure 6:**
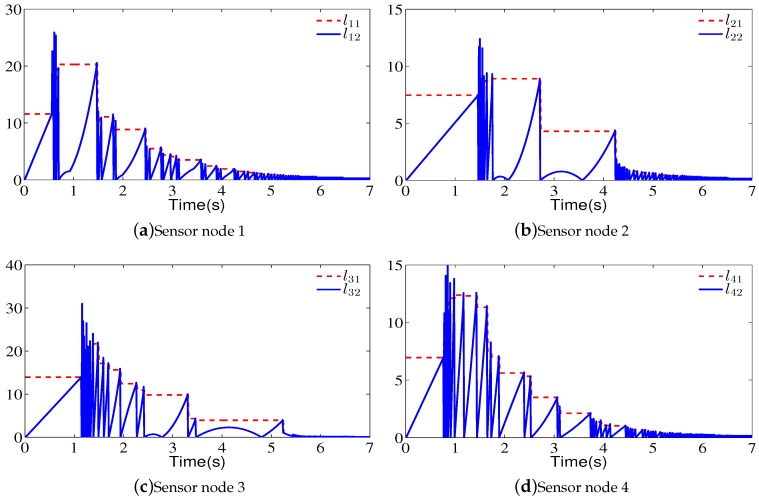
Evolution of $l_{i1}$ and $l_{i2}$, i=1,2,3,4, for the four sensor nodes.

Figure 5a shows the velocity state curves of the four sensor nodes, and Figure 5b shows the state curves on velocity inconsistency for four sensor nodes. Figure 5c shows the position state curves of the four sensor nodes, and Figure 5d shows the state curves on position inconsistency for four sensor nodes. Note that when an event is triggered, the states of the sensor nodes are broadcasted and the control signals of sensor nodes are updated. From Figure 5, one can see that the velocities and positions of sensor nodes can reach consensus. Figure 6 shows the evolution of $l_{i1}$ and $l_{i2}$ for four sensor nodes. From this figure, one can see that when an event is triggered, $h(|\beta y_{i}\left(t_{s}^{i}\right)|+|\gamma z_{i}\left(t_{s}^{i}\right)|)$ is updated. The communication frequencies of sensor node 1, sensor node 2, sensor node 3, and sensor node 4 are 14.42%, 12.28%, 18.57%, and 14.28%, respectively. Note that the data sent by sensor node 2, sensor node 3, and sensor node 4 cannot be received by other sensor nodes. Similarly, the updating frequencies of control input for sensor node 1, sensor node 2, sensor node 3, and sensor node 4 are 14.42%, 12.28%, 18.57%, and 14.28%, respectively.

## 4. Environmental Monitoring

In this section, the proposed cooperative control scheme (CCS) is applied to complete the environment monitoring.

### 4.1. Simulation Environment

**Figure 7:**
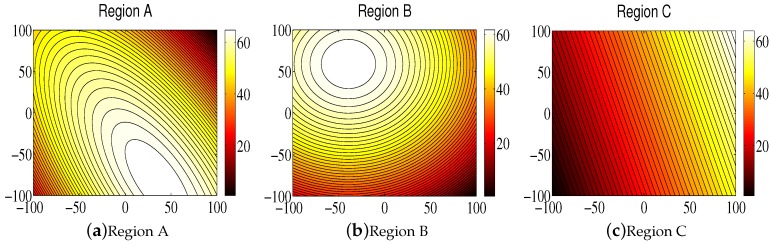
The three simulation environments.

Contour maps have been used for the simulation of the monitoring environment in recent references [2,50,51,52]. As can be seen from these references, the simulation environment is a static, scalar field. The environmental attributes include odor concentration, temperature, salinity, pH values, and so on. Its distribution can be generated according to some complex functions. We used the shifted Schwefel’s function in reference [11] to simulate the monitored environment known as Region A, the shifted sphere function in reference [11] to simulate Region B, and Schwefel’s function 2.6 from reference [11] to simulate Region C. The three regions are shown in Figure 7, from which one can see that, as the function value increases, the color gradually becomes lighter.

It should be noted that the purpose of environmental monitoring is to establish an environmental model to show the data distributions of attributes in order to provide services for environmental protection. Therefore, in order for the established model to reflect the actual distribution of the environmental attributes, we needed to use the proposed cooperative control scheme to coordinate the mobile sensor network such that it could locate the scalar field with the greatest amount of information. Therefore, from the above description, it can be seen that, in the following study, our objective was to coordinate the mobile sensor network to find the regions where color is lighter, as shown in Figure 7.

The parameters for the environmental model (3) are shown in Table 1. The mobile sensor network’s parameters are shown in Table 2. The parameters of the proposed CCS approach were β=0.2, γ=1.76, α=0.9, and h=0.1. These are different from the parameters used in the example since the chosen parameters enabled the proposed CCS approach to obtain better results for environmental monitoring. Moreover, we carefully adjusted the parameters through many simulations and then obtained the above chosen parameters. The communication topology is shown in Figure 4.

Moreover, the event-triggered finite-time (ETFT) approach described in reference [11], which is a typical comparison approach, has been used in mobile sensor networks for environmental monitoring. The corresponding parameters can be found in reference [11]. Notice that the parameters β, γ, α of the proposed CCS approach are the same with the ones for the ETFE approach.

### 4.2. Performance Metrics

Two performance metrics were used to evaluate the proposed cooperative control scheme. The first performance metric was the communication frequency, defined by

(11) $$\begin{array}{ccc} {fre}_{i} & = & \frac{Communication\;Number}{Total\;Sampling\;Number}\times 100\% \end{array}$$

where $fre_{i}$ represents the communication frequency for the ith sensor node. The “TotalSamplingNumber” represents the total number of sampling times during a run. The “CommunicationNumber” is the real communication time for the ith sensor node. Therefore, $fre_{i}$ could be used to evaluate the sensor node’s communication and computational burden.

The root-mean-square ($RMS_{i}$) error was used to evaluate the modeling accuracy.

(12) $$\begin{array}{ccc} RMS_{i} & = & \sqrt{\frac{\sum_{l=1}^{n}{\left(r_{l}\left(x_{l}\right)-q_{i}\left(x_{l}\right)\right)}^{2}}{n}} \end{array}$$

where $r_{l}\left(x_{l}\right)$ is the actual detected value for the lth sensor node at position $x_{l}$; $q_{i}\left(x_{l}\right)$ is the estimated value calculated from the environmental model for position $x_{l}$.

Based on $RMS_{i}$, another performance metric is the modeling error (ME) given by

(13) $$\begin{array}{ccc} ME & = & \frac{\sum_{l=1}^{n}\sqrt{\frac{\sum_{j=1}^{\Pi }{\left(r\left(x_{j}\right)-q_{l}\left(x_{j}\right)\right)}^{2}}{\Pi }}}{n} \end{array}$$

where $x_{j}$ is the position of the jth sampling point in the search regions, and Π is the number of total sampling points in the search regions for each environmental model. The performance metric ME was used to evaluate the accuracy of the model given by mobile sensor networks.

### 4.3. Environmental Monitoring for Region A

**Figure 8:**
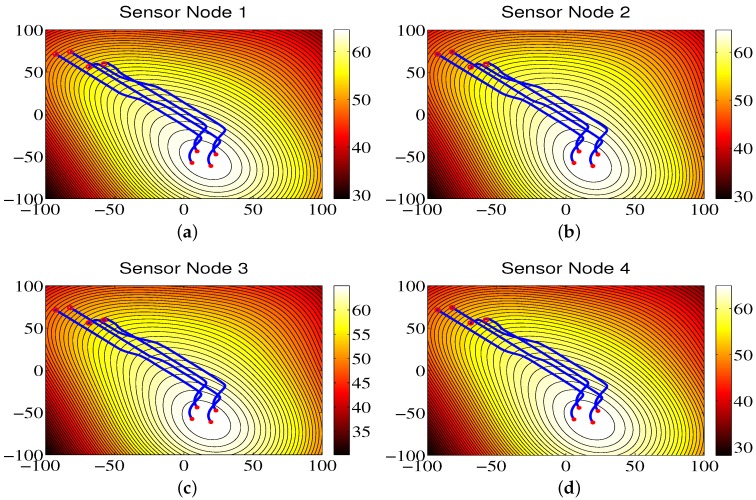
The contour maps of the environmental model built by sensor nodes for Region A.

**Figure 9:**
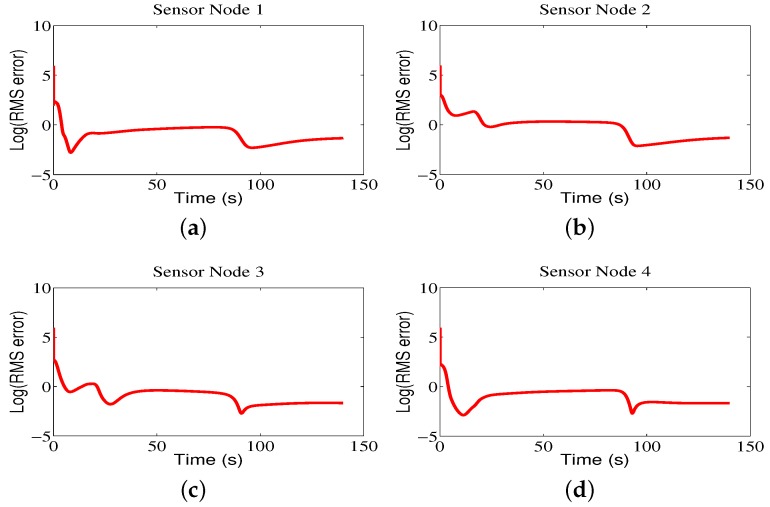
The root-mean-square (RMS) errors of sensor nodes for Region A.

Figure 8 shows the contour maps built by sensor nodes 1, 2, 3, and 4 for Region A in one run. From Figure 8, one can see that the mobile sensor network can locate the maximum values of environmental attributes where the red circles represent the start positions, the red stars represent the end positions, and the blue lines refer to the movement trajectories of sensor nodes. The contour maps provided by four sensor nodes are similar due to the directed communication topology and orderly movement. Correspondingly, the RMS errors are shown in Figure 9, from which one can clearly see that, with the movement of sensor nodes, the RMS errors become smaller.

The communication frequencies of sensor node 1, sensor node 2, sensor node 3, and sensor node 4 are shown in Table 3 for a total run time of 140 s and a sampling time of 0.01 s. From this table, one can see that, since the comparison algorithm ETFT [11] uses the continuous-time communication mechanism, the communication frequency is 100% for all sensor nodes. However, the ETFT algorithm obtains a better updating frequency, except for sensor node 1, compared with the proposed CCS approach shown in Table 4. Note that the updating frequency of the proposed CCS approach is the same as its communication frequency due to the design fashion of the proposed CCS approach. The modeling errors (ME) are shown in Table 5, from which one can see that the modeling errors for the CCS are smaller than the ones for ETFT approach [11].

### 4.4. Environmental Monitoring for Region B

**Figure 10:**
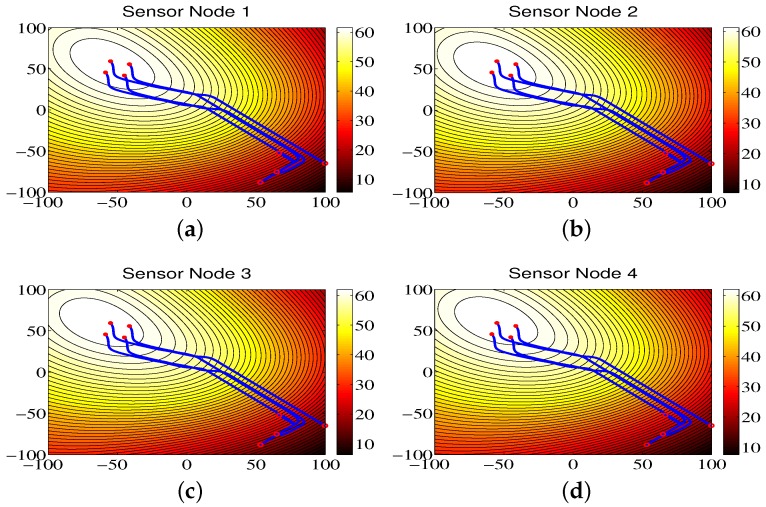
The contour maps of the environmental model built by sensor nodes for Region B.

**Figure 11:**
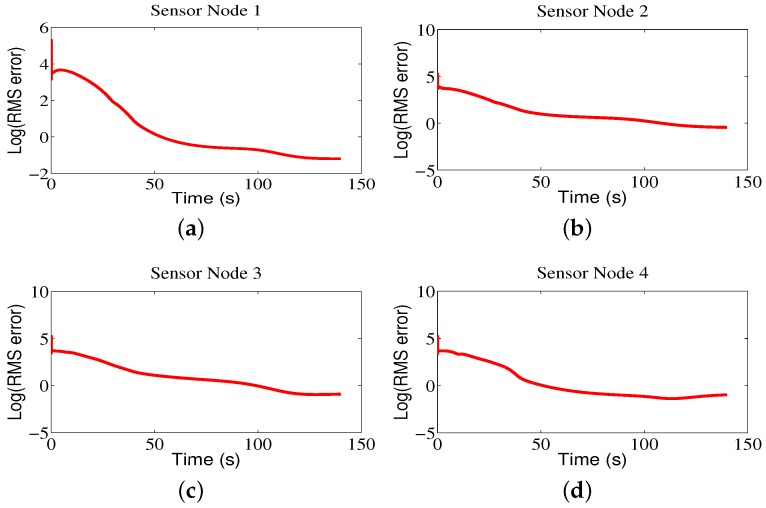
The RMS errors of sensor nodes for Region B.

The trajectories of the mobile sensor network for Region B are shown in Figure 10. As we can see from the figure, the mobile sensor network can accurately locate the brighter area in the monitored environment, and the contour color map constructed by the sensor nodes can represent the data distribution of environmental attributes. Also, it can be seen that the RMS errors are small in Figure 11.

The communication frequencies of sensor nodes are shown in Table 6 for the total run time of 140 s and the sampling time of 0.01 s. Similar to Region A, communication frequencies are also smaller compared with the ones from the ETFT approach since the ETFT approach uses the continuous-time communication mechanism. Table 7 shows the updating frequency of the control input for the two approaches where the updating frequencies of control input for the CCS approach are bigger than the ones for the ETFT approach. In addition, the modeling errors are shown in Table 8, from which one can see that the proposed CCS approach has less errors compared with the ETFT approach.

### 4.5. Environmental Monitoring for Region C

**Figure 12:**
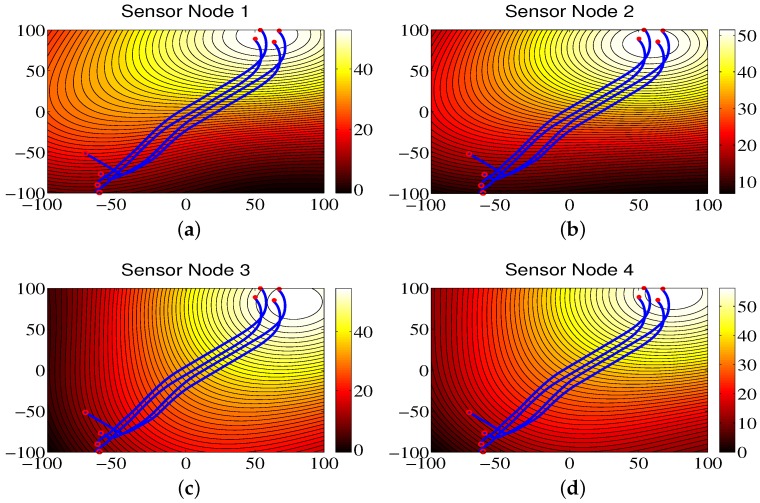
The contour maps of the environmental model built by sensor nodes for Region C.

**Figure 13:**
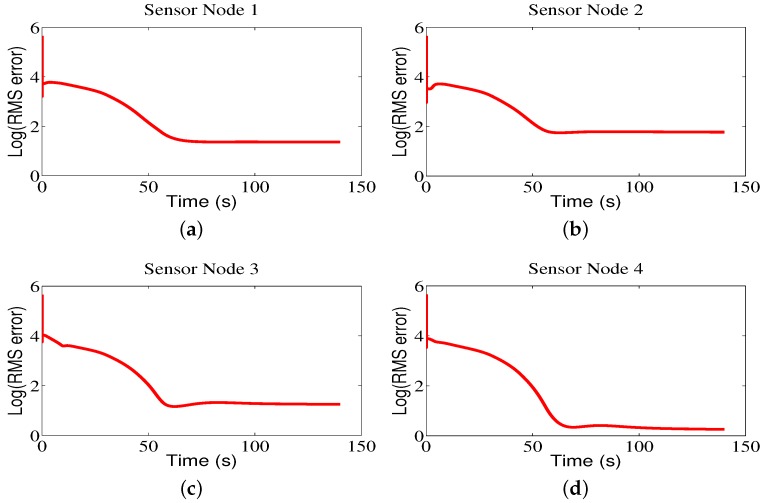
The RMS errors of sensor nodes for Region C.

Similarly, for Region C, based on the proposed CCS approach, the sensor nodes trace the maximum values of environmental attributes and produce the brighter region shown in Figure 12. In addition, the RMS errors become small with the movement of sensor nodes, as shown in Figure 13.

The communication frequencies of controllers of sensor nodes are shown in Table 9 for the total run time of 140 s and sampling time of 0.01 s, which means that the communication burden can be relaxed. On the other hand, the updating frequencies of control input for the proposed CCS approach are higher than the ones for the ETFT approach from Table 10. Moreover, Table 11 shows the modeling errors for the proposed CCS approach compared with the ETFT approach.

### 4.6. Discussion

When using finite-time consensus control, from the aforementioned results, one can see that the mobile sensor network can quickly adjust movement trajectories to track the gradient direction provided by environmental models. Compared with the original contour maps, the established environment maps with small RMS errors can better reflect the data distribution of environmental attributes. Moreover, compared with the ETFT approach, the proposed CCS approach obtains smaller modeling errors, which implies that the proposed CCS approach is efficient for building the environmental model.

Furthermore, since the event-based communication rule is used, the proposed CCS approach not only obtains a low communication frequency, but also has a lower updating frequency of control input, which implies that energy consumption of sensor nodes is saved. Even though the ETFT approach obtains a lower updating frequency of control input compared with the proposed CCS approach, the approach still needs continuous-time communication. Hence, from the aforementioned results, one can see that the proposed CCS approach shows good performance capabilities for the energy consumption and communication bandwidth of sensor nodes. It should be pointed out that one main reason for obtaining the smaller updating frequencies of control input for both the proposed CCS approach and the ETFT approach is that the gradient information from the environmental model can better guide the movement of sensor nodes toward the regions with the greatest amount of information for Region A, Region B, and Region C.

## 5. Conclusions

We designed a cooperative control scheme for the problem of environmental monitoring which includes three modules: a wireless communication module, a direction decision module, and a motion control module. In the wireless communication module, we proposed an event-based communication rule that can adjudge whether or not the data need to be transmitted. The use of the proposed event-based communication rule can save the communication bandwidth and energy consumption of sensor nodes. In the direction decision module, we used a radial basis function network to model the environmental attributes and showed the data distribution for environmental protection. In the motion control module, we designed a finite-time consensus controller that can enable the sensor nodes to quickly adjust the movement direction based on the information from the environmental model. In particular, the proposed cooperative control scheme is still efficient under the directed communication situation. Finally, we showed the effectiveness of the proposed cooperative control scheme for the problem of environmental monitoring.

## Figures and Tables

**Table 1:** Parameters of the environmental model (3).

Parameters	Values
The number of radial basis functions, *m*	20
The center, $\omega_{j}$	[−100,100]×[−100,100]
The width, $\sigma_{j}$	[80,130]
The initial weight, $\theta_{j}$	[1,5]
The initial covariance matrix, P(0)	3$I_{m}$

**Table 2:** Parameters of the mobile sensor network.

Parameters	Values
Sampling time	0.01 s
Noise leve, l *W*	3
Total run time	140 s for Region A, Region B, and Region C
The number of sensor nodes, *n*	4
The velocity range of sensor nodes	[−3 m/s, 3 m/s]

**Table 3:** Mean (standard deviation) results for the communication frequency, $fre_{i}$ (%), based on 30 runs for Region A.

Nodes	CCS (This Paper)	ETFT [11]
Sensor Node 1	3.46 (0.80)	100 (0)
Sensor Node 2	10.72 (5.81)	100(0)
Sensor Node 3	11.02 (5.29)	100(0)
Sensor Node 4	10.79 (6.07)	100(0)

**Table 4:** Mean (standard deviation) results for the updating frequency of control input (%) based on 30 runs for Region A.

Nodes	CCS (This Paper)	ETFT [11]
Sensor Node 1	3.46 (0.80)	4.66 (0.67)
Sensor Node 2	10.72 (5.81)	4.98 (2.83)
Sensor Node 3	11.02 (5.29)	4.27 (1.91)
Sensor Node 4	10.79 (6.07)	4.55 (2.46)

**Table 5:** Simulation results in the modeling error based on 30 runs for Region A.

Region	ME	CCS (This Paper)	ETFT [11]
Region A	mean	88.77	92.57
	std	31.96	57.38

**Table 6:** Mean (standard deviation) results for the communication frequency $fre_{i}$ (%) based on 30 runs for Region B.

Nodes	CCS (This Paper)	ETFT [11]
Sensor Node 1	3.20 (0.66)	100(0)
Sensor Node 2	9.75 (6.64)	100(0)
Sensor Node 3	10.42 (6.29)	100(0)
Sensor Node 4	10.49 (6.19)	100(0)

**Table 7:** Mean (standard deviation) results in the updating frequency of control input (%) based on 30 runs for Region B.

Nodes	CCS (This Paper)	ETFT [11]
Sensor Node 1	3.20 (0.66)	2.16 (0.35)
Sensor Node 2	9.75 (6.64)	1.37 (1.19)
Sensor Node 3	10.42 (6.29)	1.51 (1.10)
Sensor Node 4	10.49 (6.19)	1.62 (1.35)

**Table 8:** Simulation results in the modeling error based on 30 runs for Region B.

Region	ME	CCS (This Paper)	ETFT [11]
Region B	mean	62.02	65.31
	std	29.55	31.60

**Table 9:** Mean (standard deviation) results in the communication frequency, $fre_{i}$ (%), based on 30 runs for Region C.

Nodes	CCS (This Paper)	ETFT [11]
Sensor Node 1	4.69 (1.01)	100(0)
Sensor Node 2	4.49 (3.73)	100(0)
Sensor Node 3	4.15 (4.27)	100(0)
Sensor Node 4	3.68 (4.11)	100(0)

**Table 10:** Mean (standard deviation) results in the updating frequency of control input (%) based on 30 runs for Region C.

Nodes	CCS (This Paper)	ETFT [11]
Sensor Node 1	4.69 (1.01)	1.69 (0.38)
Sensor Node 2	4.49 (3.73)	2.12 (1.84)
Sensor Node 3	4.15 (4.27)	1.95 (1.67)
Sensor Node 4	3.68 (4.11)	2.10 (2.06)

**Table 11:** Simulation results for the modeling error based on 30 runs for Region C.

Region	ME	CCS (This Paper)	ETFT [11]
Region C	mean	93.20	100.44
	std	34.18	47.17

## Appendix A. Model Transformation

In order to prove Theorem 1, we assumed ${\bar{v}}_{i}\left(t\right)=v_{i}\left(t\right)-v_{0}$, $t\in [t_{s}^{i},t_{s+1}^{i})$ and ${\bar{x}}_{i}\left(t\right)=x_{i}\left(t\right)-x_{0}\left(t\right)$ to transform the mathematical model of the mobile sensor network. Thus, the mobile sensor network (6) with finite-time controller (8) was be changed to the following form:

(A1) $$\begin{array}{c} \left\{\begin{array}{c} {\dot{\bar{x}}}_{i}\left(t\right)={\bar{v}}_{i}\left(t\right)\\ {\dot{\bar{v}}}_{i}\left(t\right)=\mathrm{sig}(\sum_{j=0}^{n}p_{ij}\beta \left({\bar{x}}_{j}\left(t\right)-{\bar{x}}_{i}\left(t\right)\right)+\sum_{j=0}^{n}p_{ij}\beta \left(e_{jx}\left(t\right)-e_{ix}\left(t\right)\right)\\ +\sum_{j=0}^{n}p_{ij}\gamma \left({\bar{v}}_{j}\left(t\right)-{\bar{v}}_{i}\left(t\right)\right)+\sum_{j=0}^{n}p_{ij}\gamma \left(e_{jv}\left(t\right)-e_{iv}\left(t\right)\right){)}^{\alpha }\\ +\sum_{j=0}^{n}p_{ij}\beta \left({\bar{x}}_{j}\left(t\right)-{\bar{x}}_{i}\left(t\right)\right)+\sum_{j=0}^{n}p_{ij}\beta \left(e_{jx}\left(t\right)-e_{ix}\left(t\right)\right)\\ +\sum_{j=0}^{n}p_{ij}\gamma \left({\bar{v}}_{j}\left(t\right)-{\bar{v}}_{i}\left(t\right)\right)+\sum_{j=0}^{n}p_{ij}\gamma \left(e_{jv}\left(t\right)-e_{iv}\left(t\right)\right) \end{array}\right. \end{array}$$

Furthermore, in order to simplify the model presentation, we set

$$\begin{array}{c} y_{i}\left(t\right)=\sum_{j=0}^{n}p_{ij}\left({\bar{x}}_{j}\left(t\right)-{\bar{x}}_{i}\left(t\right)\right),\\ z_{i}\left(t\right)=\sum_{j=0}^{n}p_{ij}\left({\bar{v}}_{j}\left(t\right)-{\bar{v}}_{i}\left(t\right)\right)\\ e_{i}^{x}\left(t\right)=\sum_{j=0}^{n}p_{ij}\left(e_{jx}\left(t\right)-e_{ix}\left(t\right)\right),\\ e_{i}^{v}\left(t\right)=\sum_{j=0}^{n}p_{ij}\left(e_{jv}\left(t\right)-e_{iv}\left(t\right)\right) \end{array}$$

Note that $y_{i}\left(t_{s}^{i}\right)=y_{i}\left(t\right)+e_{i}^{x}\left(t\right)$ and $z_{i}\left(t_{s}^{i}\right)=z_{i}\left(t\right)+e_{i}^{v}\left(t\right)$. Let $y\left(t\right)={\left[y_{1}\left(t\right),\ldots ,y_{n}\left(t\right)\right]}^{T}$, $z\left(t\right)={\left[z_{1}\left(t\right),\ldots ,z_{n}\left(t\right)\right]}^{T}$, $e^{x}\left(t\right)={\left[e_{1}^{x}\left(t\right),\ldots ,e_{n}^{x}\left(t\right)\right]}^{T}$, and $e^{v}\left(t\right)={\left[e_{1}^{v}\left(t\right),\ldots ,e_{n}^{v}\left(t\right)\right]}^{T}$. Based on previous descriptions, the dynamics of the mobile sensor network was derived by

(A2) $$\begin{array}{c} \left\{\begin{array}{c} \dot{y}\left(t\right)=z\left(t\right)\\ \dot{z}\left(t\right)=-M\mathrm{sig}{\left(\phi \left(t\right)\right)}^{\alpha }-M\phi \left(t\right)\\ \phi \left(t\right)=\beta y\left(t\right)+\gamma z\left(t\right)+\beta e^{x}\left(t\right)+\gamma e^{v}\left(t\right) \end{array}\right. \end{array}$$

## Appendix B. Some Lemmas from References

We present Lemma 1 from reference [53], which shows the conditions of finite-time convergence. Lemma 1 can be used to prove the finite-time consensus of sensors’ state under the proposed event-based communication and finite-time consensus control approach.

**Lemma** **1.**
*For the system $\dot{x}\left(t\right)=f\left(x\left(t\right)\right)$ where $x\in N\subseteq R^{n}$ and N is the origin’s open neighborhood. If there is a continuously differentiable function V:N→R which satisfies*

$$\begin{array}{c} V(x(t))>0\\ \dot{V}\left(x\left(t\right)\right)+kV{\left(x\left(t\right)\right)}^{\alpha }<0 \end{array}$$

*where k is a positive constant and α∈(0,1), the system $\dot{x}\left(t\right)=f\left(x\left(t\right)\right)$ is finite-time stable. T:N∖{0}→(0,∞) is the map where N∖{0} is an open neighborhood which does not contain the origin. The settling time, T(x(0)), satisfies*

$$\begin{array}{c} T\left(x\left(0\right)\right)\leq \frac{1}{k(1-\alpha )}V{\left(x\left(0\right)\right)}^{1-\alpha }. \end{array}$$

Lemma 2 and Lemma 3 are from reference [15] and provide some inequalities that can be used to simply the proof process of Theorem 1.

**Lemma** **2.**
*For $p_{i}$, i=1,…,n and 0<b≤1, the inequality $(\sum_{i=1}^{n}|p_{i}{\left|\right)}^{b}\leq \sum_{i=1}^{n}|p_{i}{|}^{b}\leq n^{1-b}(\sum_{i=1}^{n}|p_{i}{\left|\right)}^{b}$ holds.*

**Lemma** **3.**
*Consider p∈R, q∈R. When the conditions c>0 and d>0 are satisfied, we have ${\left|p\right|}^{c}{\left|q\right|}^{d}\leq \frac{c}{c+d}{\left|p\right|}^{c+d}+\frac{d}{c+d}{\left|q\right|}^{c+d}$.*

## Appendix C. Some Proposed Lemmas

The inequality $\epsilon^{T}M\mathrm{sig}{\left(\epsilon \right)}^{\alpha }\geq k\sum_{i=1}^{n}{\left|\epsilon_{i}\right|}^{\alpha +1}\geq 0$ is important to simplify the proof of Theorem 1. Lemma 4 shows the structure conditions of communication topology such that the above the inequality is satisfied.

**Lemma** **4.**
*Let $\epsilon ={\left[\epsilon_{1},\ldots ,\epsilon_{n}\right]}^{T}\in R^{n}$. $I_{n}$ represent the n×n identity matrix. 0<α≤1 and $M=L_{G_{n}}+\mathrm{diag}\left\{p_{10},\ldots ,p_{n0}\right\}$. If a positive constant $k=\min_{i=1,\ldots ,n}\left\{\frac{\alpha }{\alpha +1}\left(\sum_{j=1}^{n}p_{ij}-\sum_{j=1}^{n}p_{ji}\right)+p_{i0}\right\}$ exists, where $p_{ij}$ is the non-negative adjacency elements for weighted adjacency matrix $P=[p_{ij}]$, then $\epsilon^{T}M\mathrm{sig}{\left(\epsilon \right)}^{\alpha }\geq k\sum_{i=1}^{n}{\left|\epsilon_{i}\right|}^{\alpha +1}\geq 0$.*

**Proof.** Consider $\epsilon^{T}M\mathrm{sig}{\left(\epsilon \right)}^{\alpha }$ as

$$\begin{array}{cc} -\epsilon^{T}M\mathrm{sig}{\left(\epsilon \right)}^{\alpha }= & \sum_{i=1}^{n}\epsilon_{i}\sum_{j=1}^{n}p_{ij}\left(\mathrm{sig}{\left(\epsilon_{j}\right)}^{\alpha }-\mathrm{sig}{\left(\epsilon_{i}\right)}^{\alpha }\right)-\sum_{i=1}^{n}a_{i0}{\left|\epsilon_{i}\right|}^{\alpha +1}\\ & =\sum_{i=1}^{n}\sum_{j=1}^{n}p_{ij}\epsilon_{i}\mathrm{sig}{\left(\epsilon_{j}\right)}^{\alpha }-\sum_{i=1}^{n}\left(\sum_{j=1}^{n}p_{ij}+p_{i0}\right){\left|\epsilon_{i}\right|}^{\alpha +1}. \end{array}$$

From Lemma 3, we obtain

$$\begin{array}{cc} \sum_{i=1}^{n}\sum_{j=1}^{n}p_{ij}\epsilon_{i}\mathrm{sig}{\left(\epsilon_{j}\right)}^{\alpha }\leq & \sum_{i=1}^{n}\sum_{j=1}^{n}p_{ij}\left(\frac{1}{1+\alpha }|\epsilon_{i}{|}^{\alpha +1}+\frac{\alpha }{1+\alpha }{\left|\epsilon_{j}\right|}^{\alpha +1}\right). \end{array}$$

Then, we can derive

$$\begin{array}{cc} -\epsilon^{T}M\mathrm{sig}{\left(\epsilon \right)}^{\alpha } & \leq \sum_{i=1}^{n}\sum_{j=1}^{n}p_{ij}\left(\frac{1}{1+\alpha }|\epsilon_{i}{|}^{\alpha +1}+\frac{\alpha }{1+\alpha }{\left|\epsilon_{j}\right|}^{\alpha +1}\right)\\ & -\sum_{i=1}^{n}\left(\sum_{j=1}^{n}p_{ij}+p_{i0}\right){\left|\epsilon_{i}\right|}^{\alpha +1}\\ & =\frac{\alpha }{1+\alpha }\sum_{j=1}^{n}\sum_{i=1}^{n}p_{ji}|\epsilon_{i}{|}^{\alpha +1}-\sum_{i=1}^{n}\left(\frac{\alpha }{1+\alpha }\sum_{j=1}^{n}p_{ij}+p_{i0}\right){\left|\epsilon_{i}\right|}^{\alpha +1}\\ & =-\sum_{i=1}^{n}\left(\frac{\alpha }{1+\alpha }\left(\sum_{j=1}^{n}p_{ij}-\sum_{j=1}^{n}p_{ji}\right)+p_{i0}\right){\left|\epsilon_{i}\right|}^{\alpha +1}. \end{array}$$

It is straightforward to see that $\epsilon^{T}M\mathrm{sig}{\left(\epsilon \right)}^{\alpha }\geq k\sum_{i=1}^{n}{\left|\epsilon_{i}\right|}^{\alpha +1}$. In addition, because $k=\min_{i=1,\ldots ,n}\frac{\alpha }{1+\alpha }\left(\sum_{j=1}^{n}p_{ij}-\sum_{j=1}^{n}p_{ji}\right)+p_{i0})$ is a positive constant, $\epsilon^{T}M\mathrm{sig}{\left(\epsilon \right)}^{\alpha }\geq k\sum_{i=1}^{n}{\left|\epsilon_{i}\right|}^{\alpha +1}\geq 0$. ☐

Lemma 5 and Lemma 6 describe the relationship of the given variables $\phi_{i}\left(t\right)$, $y_{i}\left(t_{s}^{i}\right)$ and $z_{i}\left(t_{s}^{i}\right)$ in (A2) and are used to simplify the proof of Theorem 1.

**Lemma** **5.**
*Consider the mobile sensor network (A2). If $\phi_{i}\left(t\right)=0$, $t\in [t_{s}^{i},t_{s+1}^{i})$, for ∀i=1,…,n, then $y_{i}\left(t_{s}^{i}\right)=0$ and $z_{i}\left(t_{s}^{i}\right)=0$, i=1,…,n.*

**Proof.** If $\phi_{i}\left(t\right)=0$, $t\in [t_{s}^{i},t_{s+1}^{i})$, ∀i=1,…,n, and $y_{i}\left(t_{s}^{i}\right)$ and $z_{i}\left(t_{s}^{i}\right)$ are nonzero for some indexes (*i*) before consensus is achieved. Since $\phi_{i}\left(t\right)=0$ ∀i=1,…,n, we obtain $\dot{z}\left(t\right)=0$ from (A2) and ${\dot{\bar{v}}}_{i}\left(t\right)=0$ from (A1). This means that ${\dot{v}}_{i}\left(t\right)=0$, ${\dot{z}}_{i}\left(t\right)=0$, and ${\dot{e}}^{v}\left(t\right)=0$. In addition, we get $\phi \left(t\right)=\beta y\left(t\right)+\gamma z\left(t\right)+\beta e^{x}\left(t\right)+\gamma e^{v}\left(t\right)=0$ from (A2). Hence, $\beta \dot{y}\left(t\right)+\gamma \dot{z}\left(t\right)+\beta {\dot{e}}^{x}\left(t\right)+\gamma {\dot{e}}^{v}\left(t\right)=0$, which implies that $\beta z\left(t\right)+\beta e^{v}\left(t\right)=0$. Hence, $\beta z(t_{s}^{i})=0$. On the other hand, since we have $\phi_{i}\left(t\right)=\beta y_{i}\left(t_{s}^{i}\right)+\gamma z_{i}\left(t_{s}^{i}\right)=0$, which implies that $y_{i}\left(t_{s}^{i}\right)=0$, this contradicts that $y_{i}\left(t_{s}^{i}\right)$ and $z_{i}\left(t_{s}^{i}\right)$ are nonzero for some indexes (*i*). The proof of this lemma is completed. ☐

**Lemma** **6.**
*Consider the mobile sensor network (A2). There is a positive constant ($k_{1}$) which establishes the following inequality:*

$$\begin{array}{c} \sum_{i=1}^{n}\phi_{i}{\left(t\right)}^{2}\geq k_{1}\sum_{i=1}^{n}\left(\beta^{2}y_{i}{\left(t_{s}^{i}\right)}^{2}+\gamma^{2}z_{i}{\left(t_{s}^{i}\right)}^{2}\right). \end{array}$$

**Proof.** Since $\phi_{i}\left(t\right)=\beta y_{i}\left(t_{s}^{i}\right)+\gamma z_{i}\left(t_{s}^{i}\right)=\left[1\;1\right]\varepsilon_{i}\left(t\right)$ where $\varepsilon_{i}\left(t\right)={\left[\beta y_{i}\left(t_{s}^{i}\right)\;\gamma z_{i}\left(t_{s}^{i}\right)\right]}^{T}$, we have

$$\begin{array}{cc} \phi_{i}{\left(t\right)}^{2} & =\varepsilon_{i}{\left(t\right)}^{T}\left(\begin{array}{cc} 1 & 1\\ 1 & 1 \end{array}\right)\varepsilon_{i}\left(t\right). \end{array}$$

Let $\Delta =\left(\begin{array}{cc} 1 & 1\\ 1 & 1 \end{array}\right)$ and the corresponding eigenvalues are ${\overset{˘}{\lambda }}_{1}=0$ and ${\overset{˘}{\lambda }}_{2}=2$, respectively. It is clear to see that Δ is a semi-positive definite matrix. Since $\sum_{i=1}^{n}\phi_{i}{\left(t\right)}^{2}=\sum_{i=1}^{n}\varepsilon_{i}{\left(t\right)}^{T}\Delta \varepsilon_{i}\left(t\right)=\zeta {\left(t\right)}^{T}I_{n}⊗\Delta \zeta \left(t\right)$, by Lemma 5, $\zeta {\left(t\right)}^{T}I_{n}⊗P\zeta \left(t\right)>0$ if ζ(t)≠0, where $\zeta \left(t\right)={\left[\varepsilon_{1}{\left(t\right)}^{T},\ldots ,\varepsilon_{n}{\left(t\right)}^{T}\right]}^{T}$. Consider the set $U=\{\sigma \in R^{2n}:\sigma^{T}\sigma =1\}$ and this set is bounded and closed. For $\frac{\zeta (t)}{{\left||\zeta \left(t\right)|\right|}_{2}}\in U$, we set $k_{1}=\min_{\frac{\zeta (t)}{{∥\zeta \left(t\right)∥}_{2}}\in U}{\left(\frac{\zeta (t)}{{∥\zeta \left(t\right)∥}_{2}}\right)}^{T}I_{n}⊗P\frac{\zeta (t)}{{∥\zeta \left(t\right)∥}_{2}}$ which exists and is larger than zero. Therefore, we obtain

$$\begin{array}{ccc} \sum_{i=1}^{n}\phi_{i}{\left(t\right)}^{2} & \geq & k_{1}{\left||\zeta \left(t\right)|\right|}_{2}^{2}\\ & & =k_{1}\sum_{i=1}^{n}\left(\beta^{2}y_{i}{\left(t_{s}^{i}\right)}^{2}+\gamma^{2}z_{i}{\left(t_{s}^{i}\right)}^{2}\right). \end{array}$$

The proof of this lemma is completed. ☐

Under the proposed event-based rule (2) for the mobile sensor network (A2), we show an inequality condition that is used to simplify the proof of Theorem 1.

**Lemma** **7.**
*Consider the mobile sensor network (A2) using the event-based rule (2). The following inequality holds:*

$$\begin{array}{c} {\left(\beta e^{x}\left(t\right)+\gamma e^{v}\left(t\right)\right)}^{T}M\mathrm{sig}{\left(\phi \left(t\right)\right)}^{\alpha }\leq \chi \sum_{i=1}^{n}{\left|\phi_{i}\left(t\right)\right|}^{\alpha +1} \end{array}$$

*where $\chi =\frac{2h}{\sqrt{k_{1}}}∥M∥n^{\frac{1-\alpha }{2}}$ and $k_{1}$ is a positive constant.*

**Proof.** We can obtain

$$\begin{array}{ccc} {\left(\beta e^{x}\left(t\right)+\gamma e^{v}\left(t\right)\right)}^{T}M\mathrm{sig}{\left(\phi \left(t\right)\right)}^{\alpha } & \leq & ∥\beta e^{x}\left(t\right)+\gamma e^{v}{\left(t\right)∥∥M∥∥|\phi \left(t\right)|}^{\alpha }∥. \end{array}$$

Further, we have

$$\begin{array}{ccc} ∥\beta e^{x}\left(t\right)+\gamma e^{v}\left(t\right)∥ & \leq & ∥M\left(\beta e_{x}\left(t\right)+\gamma e_{v}\left(t\right)\right)∥+∥D\left(\beta e_{0x}\left(t\right)+\gamma e_{0v}\left(t\right)\right)∥\\ & & \leq ∥M∥∥\beta e_{x}\left(t\right)+\gamma e_{v}\left(t\right)∥+∥D\left(\beta e_{0x}\left(t\right)+\gamma e_{0v}\left(t\right)\right)∥ \end{array}$$

where $e_{x}\left(t\right)={\left[e_{1x}\left(t\right),\ldots ,e_{nx}\left(t\right)\right]}^{T}$, $e_{v}\left(t\right)={\left[e_{1v}\left(t\right),\ldots ,e_{nv}\left(t\right)\right]}^{T}$, $e_{0x}\left(t\right)={\left[e_{10x}\left(t\right),\ldots ,e_{n0x}\left(t\right)\right]}^{T}$, $e_{0v}\left(t\right)={\left[e_{10v}\left(t\right),\ldots ,e_{n0v}\left(t\right)\right]}^{T}$, and $D=\mathrm{diag}\{p_{10},\ldots ,p_{n0}\}$. Event-based rule (2) enforces the following conditions as $∥M∥|\beta e_{ix}\left(t\right)+\gamma e_{iv}\left(t\right)|+p_{i0}|\beta e_{i0x}\left(t\right)+\gamma e_{i0v}\left(t\right)|\leq h(|\beta y_{i}\left(t_{s}^{i}\right)|+|\gamma z_{i}\left(t_{s}^{i}\right)\left|\right)$ for $t\in [t_{s}^{i},t_{s+1}^{i})$, which means ${∥M∥}^{2}{\left(\beta e_{ix}\left(t\right)+\gamma e_{iv}\left(t\right)\right)}^{2}+{\left(p_{i0}\left(\beta e_{i0x}\left(t\right)+\gamma e_{i0v}\left(t\right)\right)\right)}^{2}\leq h^{2}\left(\beta^{2}y_{i}^{2}\left(t_{s}^{i}\right)+\gamma^{2}z_{i}^{2}\left(t_{s}^{i}\right)\right)$ From Lemma 6, we have ${∥M∥}^{2}∥\beta e_{x}\left(t\right)+\gamma e_{v}{\left(t\right)∥}^{2}+∥D\left(\beta e_{0x}\left(t\right)+\gamma e_{0v}\left(t\right)\right){∥}^{2}\leq \frac{h^{2}}{k_{1}}{∥\phi \left(t\right)∥}^{2}$. Further, we obtain $∥M∥∥\beta e_{x}\left(t\right)+\gamma e_{v}\left(t\right)∥+∥D\left(\beta e_{0x}\left(t\right)+\gamma e_{0v}\left(t\right)\right)∥\leq \frac{2h}{\sqrt{k_{1}}}∥\phi \left(t\right)∥$. Hence, we derive

$$\begin{array}{c} ∥\beta e^{x}\left(t\right)+\gamma e^{v}\left(t\right)∥\leq \frac{h}{\sqrt{k_{1}}}∥\phi \left(t\right)∥. \end{array}$$

Next, from Lemma 2, ${∥|\phi \left(t\right)|}^{\alpha }{∥}^{2}=\sum_{i=1}^{n}|\phi_{i}{\left(t\right)|}^{2\alpha }\leq n^{1-\alpha }(\sum_{i=1}^{n}|\phi_{i}{\left(t\right)|}^{2}{)}^{\alpha }=n^{1-\alpha }{∥\phi \left(t\right)∥}^{2\alpha }$ such that ${∥|\phi \left(t\right)|}^{\alpha }∥\leq n^{\frac{1-\alpha }{2}}{∥\phi \left(t\right)∥}^{\alpha }$. ${∥\phi \left(t\right)∥}^{\alpha +1}={\left(\sum_{i=1}^{n}\phi_{i}{\left(t\right)}^{2}\right)}^{\frac{\alpha +1}{2}}\leq \sum_{i=1}^{n}{\left|\phi_{i}\left(t\right)\right|}^{\alpha +1}$ from Lemma 2. Hence, we have

$$\begin{array}{ccc} {\left(\beta e^{x}\left(t\right)+\gamma e^{v}\left(t\right)\right)}^{T}M\mathrm{sig}{\left(\phi \left(t\right)\right)}^{\alpha } & \leq & \frac{h}{\sqrt{k_{1}}}∥M∥n^{\frac{1-\alpha }{2}}{∥\phi \left(t\right)∥}^{\alpha +1}\leq \chi \sum_{i=1}^{n}{\left|\phi_{i}\left(t\right)\right|}^{\alpha +1} \end{array}$$

where $\chi =\frac{h}{\sqrt{k_{1}}}∥M∥n^{\frac{1-\alpha }{2}}$. ☐

Lemma 8 shows the conditions of the parameters from the proposed event-based rule (2) which is used to simplify the proof of Theorem 1.

**Lemma** **8.**
*For the mobile sensor network (A2) using the event-based rule (2), if positive constants $k_{1}$ and $k_{1}>2h^{2}$ exist, the following inequality is established:*

$$\begin{array}{c} ∥\beta e^{x}\left(t\right)+\gamma e^{v}\left(t\right)∥\leq \sqrt{\frac{2h^{2}}{k_{1}-2h^{2}}}∥\beta y\left(t\right)+\gamma z\left(t\right)∥. \end{array}$$

**Proof.** We consider the event-triggered rule (2) and have ${∥M∥}^{2}∥\beta e_{x}\left(t\right)+\gamma e_{v}{\left(t\right)∥}^{2}+∥D\left(\beta e_{0x}\left(t\right)+\gamma e_{0v}\left(t\right)\right){∥}^{2}\leq \frac{h^{2}}{k_{1}}{∥\phi \left(t\right)∥}^{2}$. Moreover,

$$\begin{array}{cc} {∥\phi \left(t\right)∥}^{2} & =\sum_{i=1}^{n}{\left(\beta y_{i}\left(t\right)+\gamma z_{i}\left(t\right)+\beta e_{i}^{x}\left(t\right)+\gamma e_{i}^{v}\left(t\right)\right)}^{2}\\ & \leq \sum_{i=1}^{n}\left(2{\left(\beta y_{i}\left(t\right)+\gamma z_{i}\left(t\right)\right)}^{2}+2{\left(\beta e_{i}^{x}\left(t\right)+\gamma e_{i}^{v}\left(t\right)\right)}^{2}\right)\\ & \leq {2∥\beta y\left(t\right)+\gamma z\left(t\right)∥}^{2}+2{∥\beta e^{x}\left(t\right)+\gamma e^{v}\left(t\right)∥}^{2}\\ & \leq {2∥\beta y\left(t\right)+\gamma z\left(t\right)∥}^{2}+{2∥M∥}^{2}{∥\beta e_{x}\left(t\right)+\gamma e_{v}\left(t\right)∥}^{2}\\ & +2∥D\left(\beta e_{0x}\left(t\right)+\gamma e_{0v}\left(t\right)\right){∥}^{2}. \end{array}$$

Clearly, one can derive

$$\begin{array}{c} ∥M∥∥\beta e_{x}\left(t\right)+\gamma e_{v}\left(t\right)∥+∥D\left(\beta e_{0x}\left(t\right)+\gamma e_{0v}\left(t\right)\right)∥\leq \sqrt{\frac{2h^{2}}{k_{1}-2h^{2}}}∥\beta y\left(t\right)+\gamma z\left(t\right)∥ \end{array}$$

where $k_{1}>2h^{2}$. Since $∥\beta e^{x}\left(t\right)+\gamma e^{v}\left(t\right)∥\leq ∥M∥∥\beta e_{x}\left(t\right)+\gamma e_{v}\left(t\right)∥+∥D\left(\beta e_{0x}\left(t\right)+\gamma e_{0v}\left(t\right)\right)∥$, we get

$$\begin{array}{c} ∥\beta e^{x}\left(t\right)+\gamma e^{v}\left(t\right)∥\leq \sqrt{\frac{2h^{2}}{k_{1}-2h^{2}}}∥\beta y\left(t\right)+\gamma z\left(t\right)∥. \end{array}$$

 ☐

## Appendix D. Proof of Theorem 1

The following proof is divided into four parts. In Part I, we prove the given Lyapunov candidate function, V(t)≥0. In Part II, we prove that mobile sensor network (2) asymptotically reaches consensus. In Part III, we prove that mobile sensor network (2) reaches consensus in finite time. In Part IV, Zeno-behaviors are excluded from mobile sensor network (2) before consensus is achieved.

***Part I: We prove the given Lyapunov candidate function,*V(t)≥0.** The following Lyapunov candidate function is given as

(A3) $$\begin{array}{c} V\left(t\right)=\frac{1}{2}\xi {\left(t\right)}^{T}\left(\begin{array}{cc} \beta \gamma (M+M^{T}) & \beta I_{n}\\ \beta I_{n} & \gamma I_{n} \end{array}\right)\xi \left(t\right) \end{array}$$

where $I_{n}$ is a n-dimensional unit matrix and $\xi \left(t\right)={\left[y{\left(t\right)}^{T},z{\left(t\right)}^{T}\right]}^{T}$. From Lemma 4, we obtain that for any vector $\epsilon \in R^{n}$, $\epsilon^{T}M\epsilon \geq 0$, which means $M+M^{T}$ is a positive definite matrix.

The following matrix variable is set as

$$\begin{array}{c} \Omega =\left(\begin{array}{cc} \beta \gamma (M+M^{T}) & \beta I_{n}\\ \beta I_{n} & \gamma I_{n} \end{array}\right). \end{array}$$

As $M+M^{T}$ is a positive definite matrix, it can be rewritten as $\Gamma^{-1}\Lambda \Gamma$ where $\Lambda =\mathrm{diag}\{\mu_{1},\mu_{2},\ldots ,\mu_{n}\}$ is a diagonal matrix consisting of the eigenvalues of $M+M^{T}$. Then, we derive

$$\begin{array}{c} \Omega ={\left(\begin{array}{cc} \Gamma & 0\\ 0 & \Gamma \end{array}\right)}^{-1}\left(\begin{array}{cc} \beta \gamma \Lambda & \beta I_{n}\\ \beta I_{n} & \gamma I_{n} \end{array}\right)\left(\begin{array}{cc} \Gamma & 0\\ 0 & \Gamma \end{array}\right). \end{array}$$

The below matrix variable is derived as

$$\begin{array}{c} \bar{\Omega }=\left(\begin{array}{cc} \beta \gamma \Lambda & \beta I_{n}\\ \beta I_{n} & \gamma I_{n} \end{array}\right). \end{array}$$

Further, the eigenvalues of matrix $\bar{\Omega }$ are solved as

$$\begin{array}{cc} \det(\lambda I_{2n}-\bar{\Omega }) & =\det\left(\begin{array}{cc} \lambda I_{n}-\beta \gamma \Lambda & -\beta I_{n}\\ -\beta I_{n} & \lambda I_{n}-\gamma I_{n} \end{array}\right)\\ & =\prod_{i=1}^{n}\left[\lambda^{2}-\lambda \left(\gamma +\beta \gamma \mu_{i}\right)+\beta \gamma^{2}\mu_{i}-\beta^{2}\right]. \end{array}$$

The eigenvalues of $\bar{\Omega }$ are calculated by

$$\begin{array}{c} \lambda_{i\pm }=\frac{\gamma +\beta \gamma \mu_{i}\pm \sqrt{{\left(\gamma +\beta \gamma \mu_{i}\right)}^{2}-c}}{2} \end{array}$$

where $c=4(\beta \gamma^{2}\mu_{i}-\beta^{2})$. $\lambda_{i+}$ and $\lambda_{i-}$ are called the eigenvalues of $\bar{\Omega }$. When c>0, i.e., $\gamma >\sqrt{\frac{\beta }{\mu_{\min}}}$ where $\mu_{\min}$ is the minimum eigenvalue of $M+M^{T}$, and $\bar{\Omega }$ has positive eigenvalues. As a result, the matrix Ω is a positive definite matrix. Thus, V(t)≥0.

***Part II: We prove that mobile sensor network (2) asymptotically reaches consensus.***

We obtain the derivative of the Lyapunov candidate function (A3) for time *t* as

(A4) $$\begin{array}{ccc} \dot{V}\left(t\right) & = & y{\left(t\right)}^{T}\beta \gamma \left(M+M^{T}\right)z\left(t\right)+z{\left(t\right)}^{T}\beta I_{n}z\left(t\right)+y{\left(t\right)}^{T}\beta I_{n}\dot{z}\left(t\right)+z{\left(t\right)}^{T}\gamma I_{n}\dot{z}\left(t\right)\\ & & =-z{\left(t\right)}^{T}\left(\gamma^{2}M-\beta I_{n}\right)z\left(t\right)-y{\left(t\right)}^{T}\beta^{2}My\left(t\right)-\left(\beta y{\left(t\right)}^{T}+\gamma z{\left(t\right)}^{T}\right)M\mathrm{sig}{\left(\phi \left(t\right)\right)}^{\alpha }\\ & & -\left(\beta y{\left(t\right)}^{T}+\gamma z{\left(t\right)}^{T}\right)M\left(\beta e^{x}\left(t\right)+\gamma e^{v}\left(t\right)\right)\\ & & =-z{\left(t\right)}^{T}\left(\gamma^{2}M-\beta I_{n}\right)z\left(t\right)-y{\left(t\right)}^{T}\beta^{2}My\left(t\right)-\phi {\left(t\right)}^{T}M\mathrm{sig}{\left(\phi \left(t\right)\right)}^{\alpha }\\ & & +{\left(\beta e^{x}\left(t\right)+\gamma e^{v}\left(t\right)\right)}^{T}M\mathrm{sig}{\left(\phi \left(t\right)\right)}^{\alpha }-\left(\beta y{\left(t\right)}^{T}+\gamma z{\left(t\right)}^{T}\right)M\left(\beta e^{x}\left(t\right)+\gamma e^{v}\left(t\right)\right). \end{array}$$

One can see that $\dot{V}\left(t\right)$ consists of five items. The first item is $-z{\left(t\right)}^{T}\left(\gamma^{2}M-\beta I_{n}\right)z\left(t\right)$. The second item is $-y{\left(t\right)}^{T}\beta^{2}My\left(t\right)$. The third item is $-\phi {\left(t\right)}^{T}M\mathrm{sig}{\left(\phi \left(t\right)\right)}^{\alpha }$. The fourth item is $+{\left(\beta e^{x}\left(t\right)+\gamma e^{v}\left(t\right)\right)}^{T}M\mathrm{sig}{\left(\phi \left(t\right)\right)}^{\alpha }$. The fifth item is $-\left(\beta y{\left(t\right)}^{T}+\gamma z{\left(t\right)}^{T}\right)M\left(\beta e^{x}\left(t\right)+\gamma e^{v}\left(t\right)\right)$. In order to use the Lyapunov theory, we first deal with the third item and the fourth item.

Consider the third item; from Lemma 4, we obtain the following inequality as

(A5) $$\begin{array}{c} \phi {\left(t\right)}^{T}M\mathrm{sig}{\left(\phi \left(t\right)\right)}^{\alpha }\geq k\sum_{i=1}^{n}{\left|\phi_{i}\left(t\right)\right|}^{\alpha +1}. \end{array}$$

Consider the fourth item, by Lemma 7, we have the following inequality as

(A6) $$\begin{array}{c} {\left(\beta e^{x}\left(t\right)+\gamma e^{v}\left(t\right)\right)}^{T}M\mathrm{sig}{\left(\phi \left(t\right)\right)}^{\alpha }\leq \chi \sum_{i=1}^{n}{\left|\phi_{i}\left(t\right)\right|}^{\alpha +1}. \end{array}$$

Substituting (A5) and (A6) into (A4) leads to the following inequality as

(A7) $$\begin{array}{cc} \dot{V}\left(t\right)\leq & -z{\left(t\right)}^{T}\left(\gamma^{2}M-\beta I_{n}\right)z\left(t\right)-y{\left(t\right)}^{T}\beta^{2}My\left(t\right)-\left(k-\chi \right)\sum_{i=1}^{n}{\left|\phi_{i}\left(t\right)\right|}^{\alpha +1}\\ & +∥\beta y\left(t\right)+\gamma z\left(t\right)∥∥M∥∥\beta e^{x}\left(t\right)+\gamma e^{v}\left(t\right)∥. \end{array}$$

Since $h<\frac{k\sqrt{k_{1}}}{∥M∥n^{\frac{1-\alpha }{2}}}$, k−χ>0.

From Lemma 8, we have the following inequality as

(A8) $$\begin{array}{c} ∥\beta e^{x}\left(t\right)+\gamma e^{v}\left(t\right)∥\leq \sqrt{\frac{2h^{2}}{k_{1}-2h^{2}}}∥\beta y\left(t\right)+\gamma z\left(t\right)∥. \end{array}$$

Substituting (A8) into (A7) gives the following result:

(A9) $$\begin{array}{ccc} \dot{V}\left(t\right) & \leq & -z{\left(t\right)}^{T}\left(\gamma^{2}M-\beta I_{n}\right)z\left(t\right)-y{\left(t\right)}^{T}\beta^{2}My\left(t\right)-\left(k-\chi \right)\sum_{i=1}^{n}|\phi_{i}\left(t\right){|}^{\alpha +1}+\delta {∥\beta y\left(t\right)+\gamma z\left(t\right)∥}^{2}\\ & & \leq -z{\left(t\right)}^{T}\left(\gamma^{2}M-\beta I_{n}-2\delta \gamma^{2}I_{n}\right)z\left(t\right)-y{\left(t\right)}^{T}\left(\beta^{2}M-2\delta \beta^{2}I_{n}\right)y\left(t\right)-\left(k-\chi \right)\sum_{i=1}^{n}{\left|\phi_{i}\right|}^{\alpha +1} \end{array}$$

where $\delta =∥M∥\sqrt{\frac{2h^{2}}{k_{1}-2h^{2}}}$.

Since $\gamma >\sqrt{\frac{\beta }{\mu_{\min}}}$, $h<\min\{\frac{k\sqrt{k_{1}}}{∥M∥n^{\frac{1-\alpha }{2}}},\sqrt{\frac{k_{1}}{2}}\}$, and $\delta <\frac{\gamma^{2}\mu_{\min}-\beta }{2\gamma^{2}}$, $\dot{V}\left(t\right)\leq 0$. $\dot{V}\left(t\right)=0$ if and only if $y\left(t\right)=0_{n}$ and $z\left(t\right)=0_{n}$. Hence, the state (y(t),z(t)) converges asymptotically to $\left(0_{n},0_{n}\right)$, and it illustrates that $x_{i}\left(t\right)=x_{0}\left(t\right)$ and $v_{i}\left(t\right)=v_{0}$ for any i=1,…n.

***Part III: We prove that mobile sensor network (2) reaches consensus in finite time.***

Since $\sum_{i=1}^{n}\phi {\left(t\right)}_{i}^{2}\geq k_{1}\sum_{i=1}^{n}\left(\beta^{2}y_{i}{\left(t_{s}^{i}\right)}^{2}+\gamma^{2}z_{i}{\left(t_{s}^{i}\right)}^{2}\right)$ from Lemma 6 and the state (y(t),z(t)) can asymptotically converge to $\left(0_{n},0_{n}\right)$ in Part II, we have $\sum_{i=1}^{n}y_{i}{\left(t_{s}^{i}\right)}^{2}\geq \sum_{i=1}^{n}y_{i}{\left(t\right)}^{2}$ and $\sum_{i=1}^{n}z_{i}{\left(t_{s}^{i}\right)}^{2}\geq \sum_{i=1}^{n}z_{i}{\left(t\right)}^{2}$ for each $t\in [t_{s}^{i},t_{s+1}^{i})$, which implies

$$\begin{array}{ccc} \sum_{i=1}^{n}\phi_{i}{\left(t\right)}^{2} & \geq & k_{1}\sum_{i=1}^{n}\left(\beta^{2}y_{i}{\left(t\right)}^{2}+\gamma^{2}z_{i}{\left(t\right)}^{2}\right)\geq k_{1}\rho_{1}\sum_{i=1}^{n}\left(y_{i}{\left(t\right)}^{2}+z_{i}{\left(t\right)}^{2}\right) \end{array}$$

where $\rho_{1}=\min\left\{\beta^{2},\gamma^{2}\right\}$.

Assuming that $\lambda_{\max}$ is the maximum eigenvalue of Ω and that $V\left(t\right)\leq \frac{1}{2}\lambda_{\max}{∥\xi \left(t\right)∥}_{2}^{2}=\frac{1}{2}\lambda_{\max}\sum_{i=1}^{n}\left(y_{i}{\left(t\right)}^{2}+z_{i}{\left(t\right)}^{2}\right)\leq \frac{1}{2k_{1}\rho_{1}}\lambda_{\max}\sum_{i=1}^{n}\phi_{i}^{2}\left(t\right)$. From Lemma 2, we obtains $V{\left(t\right)}^{\frac{1+\alpha }{2}}\leq {\left(\frac{1}{2k_{1}\rho_{1}}\lambda_{\max}\right)}^{\frac{1+\alpha }{2}}\sum_{i=1}^{n}{\left|\phi_{i}\left(t\right)\right|}^{1+\alpha }$. Moreover, from (A9), we have $\dot{V}\left(t\right)\leq -\left(k-\chi \right)\sum_{i=1}^{n}{\left|\phi_{i}\left(t\right)\right|}^{\alpha +1}$. The constant $k_{2}$ is defined as

(A10) $$\begin{array}{ccc} k_{2} & = & \frac{\left(k-\chi \right)}{{\left(\frac{1}{2k_{1}\rho_{1}}\lambda_{\max}\right)}^{\frac{1+\alpha }{2}}} \end{array}$$

and we have $\dot{V}\left(t\right)+k_{2}V{\left(t\right)}^{\frac{\alpha +1}{2}}\leq 0$. By Lemma 1, $x_{i}\left(t\right)\to x_{0}\left(t\right)$ and $v_{i}\left(t\right)\to v_{0}$, i=1,…,n in finite time.

***Part IV: Zeno-behaviors are excluded from mobile sensor network (2) before consensus is achieved.***

Note that $e_{ix}\left(t_{s}^{i}\right)=0$ and $x_{i}\left(t_{s}^{i}\right)$ is a constant for $t\in [t_{s}^{i},t_{s+1}^{i})$. Hence, we derive the following inequality as

$$\begin{array}{ccc} |e_{ix}\left(t\right)| & \leq & |\int_{t_{s}^{i}}^{t}{\dot{e}}_{ix}\left(t\right)d\tau |\leq \int_{t_{s}^{i}}^{t}\left|{\dot{e}}_{ix}\left(t\right)\right|d\tau \\ & & =\int_{t_{s}^{i}}^{t}\left|{\dot{x}}_{i}\left(t\right)\right|d\tau \leq s_{v}\left(t-t_{s}^{i}\right),t\in \left[t_{s}^{i},t_{s+1}^{i}\right) \end{array}$$

where $s_{v}$ is the maximum value of the velocity’s absolute value. Similarly, we can derive

$$\begin{array}{c} |e_{iv}\left(t\right)|\leq s_{a}\left(t-t_{s}^{i}\right)\\ |e_{i0x}\left(t\right)|\leq s_{v}\left(t-t_{s}^{i}\right)\\ |e_{i0v}\left(t\right)|\leq s_{a}\left(t-t_{s}^{i}\right) \end{array}$$

where $s_{a}$ is the maximum value of the acceleration’absolute value. Moreover, we have $∥M∥|\beta e_{ix}+\gamma e_{iv}|+p_{i0}|\beta e_{i0x}+\gamma e_{i0v}|\leq ((∥M∥+1)\beta s_{v}+\left(∥M∥+1\right)\gamma s_{a})\left(t-t_{s}^{i}\right)$. In terms of the event-based rule (2), the next event is triggered at $t=t_{s+1}^{i}$, which means $∥M∥|\beta e_{ix}+\gamma e_{iv}|+p_{i0}|\beta e_{i0x}+\gamma e_{i0v}|>h(|\beta y_{i}\left(t_{s}^{i}\right)|+|\gamma z_{i}\left(t_{s}^{i}\right)\left|\right)$ at $t=t_{s+1}^{i}$. Hence, we have $((∥M∥+1)\beta s_{v}+\left(∥M∥+1\right)\gamma s_{a})\left(t_{s+1}^{i}-t_{s}^{i}\right)>h(|\beta y_{i}\left(t_{s}^{i}\right)|+|\gamma z_{i}\left(t_{s}^{i}\right)\left|\right)$. Before consensus is achieved, $t_{s+1}^{i}-t_{s}^{i}>\frac{h(|\beta y_{i}\left(t_{s}^{i}\right)|+|\gamma z_{i}\left(t_{s}^{i}\right)|)}{\left(∥M∥+1\right)\beta s_{v}+\left(∥M∥+1\right)\gamma s_{a}}>0$, which means the Zeno-behaviors are avoided.

## References

[1] Farrell J.A., Pang S., Li W. (2003). Plume mapping via hidden markov methods. IEEE Trans. Syst. Man Cybern. Part B Cybern..

[2] Leonard N.E., Paley D.A., Lekien F., Sepulchre R., Fratantoni D.M., Davis R. (2007). Collective motion, sensor networks and ocean sampling. Proc. IEEE.

[3] Lu Q., Han Q.-L., Xie X., Liu S. (2014). A finite-time motion control strategy for odor source localization. IEEE Trans. Ind. Electron..

[4] Lu Q., Liu S., Xie X., Wang J. (2013). Decision making and finite-time motion control for a group of robots. IEEE Trans. Cybern..

[5] Ren W., Beard R. (2008). Distributed Consensus in Multi-Vehicle Cooperative Control—Theory and Applications.

[6] Cao Y., Yu W., Ren W., Chen G. (2013). An overview of recent progress in the study of distributed multi-agent coordination. IEEE Trans. Ind. Inform..

[7] Ge X., Han Q.-L., Yang F. (2017). Event-based set-membership leader-following consensus of networked multi-agent systems subject to limited communication resources and unknown-but-bounded noise. IEEE Trans. Ind. Electron..

[8] He W., Qian F., Cao J. (2017). Pinning-controlled synchronization of delayed neural networks with distributed-delay coupling via impulsive control. Neural Netw..

[9] He W., Qian F., Lam J., Chen G., Han Q.-L., Kurths J. (2015). Quasi-synchronization of heterogeneous dynamic networks via distributed impulsive control: Error estimation, optimization and design. Automatica.

[10] Li Y., Tong S., Li T. (2015). Adaptive fuzzy output feedback dynamic surface control of interconnected nonlinear pure-feedback systems. IEEE Trans. Cybern..

[11] Lu Q., Han Q.-L., Zhang B., Liu D., Liu S. (2017). Cooperative control of mobile sensor networks for environmental monitoring: An event-triggered finite-time control scheme. IEEE Trans. Cybern..

[12] Wang X., Li S., Shi P. (2014). Distributed finite-time containment control for double-integrator multiagent systems. IEEE Trans. Cybern..

[13] Jiang Y., Zhang H., Chen J. (2017). Sign-consensus of linear multi-agent systems over signed directed graphs. IEEE Trans. Ind. Electron..

[14] Valcher M.E., Zorzan I. (2017). On the consensus of homogeneous multi-agent systems with positivity constraints. IEEE Trans. Autom. Control.

[15] Li S., Du H., Lin X. (2011). Finite-time consensus algorithm for multi-agent systems with double-integrator dynamics. Automatica.

[16] Lu X., Lu R., Chen S., Lu J. (2013). Finite-time distributed tracking control for multi-agent systems with a virtual leader. IEEE Trans. Circuits Syst. I Regul. Pap..

[17] Xiao F., Wang L., Chen T. (2014). Finite-time consensus in networks of integrator-like dynamic agents with directional link failure. IEEE Trans. Autom. Control.

[18] Du H., Wen G., Chen G., Cao J., Alsaadi F.E. (2017). A distributed finite-time consensus algorithm for higher-order leaderless and leader-following multiagent systems. IEEE Trans. Syst. Man Cybern. Syst..

[19] Li S., Zhou M., Yu X. (2013). Design and implementation of terminal sliding mode control method for pmsm speed regulation system. IEEE Trans. Ind. Inform..

[20] Cao Y., Ren W., Meng Z. (2010). Decentralized finite-time sliding mode estimators and their applications in decentralized finite-time formation tracking. Syst. Control Lett..

[21] Cortés J. (2006). Finite-time convergent gradient flows with applications to network consensus. Automatica.

[22] Hui Q., Haddad W.M., Bhat S.P. (2008). Finite-time semistability and consensus for nonlinear dynamical networks. IEEE Trans. Autom. Control.

[23] Hui Q. (2011). Finite-time rendezvous algorithms for mobile autonomous agents. IEEE Trans. Autom. Control.

[24] Du H., Li S., Qian C. (2011). Finite-time attitude tracking control of spacecraft with application to attitude synchronization. IEEE Trans. Autom. Control.

[25] Meng Z., Ren W., You Z. (2010). Distributed finite-time attitude containment control for multiple rigid bodies. Automatica.

[26] Wang X., Hong Y. Finite-time consensus for multi-agent networks with second-order agent dynamics. Proceedings of the 17th World Congress, The International Federation of Automatic Control.

[27] Xiao F., Wang L., Chen J., Gao Y. (2009). Finite-time formation control for multi-agent systems. Automatica.

[28] Åström K.J., Bernhardsson B. (1999). Comparison of periodic and event based sampling for first-order stochastic systems. IFAC Proc. Vol..

[29] Diaz-Cacho M., Delgado E., Barreiro A., Falcón P. (2017). Basic send-on-Delta sampling for signal tracking-error reduction. Sensors.

[30] Gao Y., Li Y., Peng L., Liu J. (2018). Design of event-triggered fault-tolerant control for stochastic systems with time-delays. Sensors.

[31] Miskowicz M. (2006). Send-on-Delta concept: An event-based data reporting strategy. Sensors.

[32] Putra P.E.S., Brusey J., Gaura E., Vesilo R. (2018). An event-triggered machine learning approach for accelerometer-based fall detection. Sensors.

[33] Suh Y.S. (2007). Send-on-Delta sensor data transmission with a linear predictor. Sensors.

[34] Xu Z., Liu G., Cheng H.Y.B., Lin F. (2017). Trail-based search for efficient event report to mobile actors in wireless sensor and actor networks. Sensors.

[35] Dimarogonas D.V., Frazzoli E., Johansson K.H. (2012). Distributed event-triggered control for multi-agent systems. IEEE Trans. Autom. Control.

[36] Ding D., Wang Z., Ho D.W.C., Wei G. (2016). Observer-based event-triggering consensus control for multiagent systems with lossy sensors and cyber-attacks. IEEE Trans. Cybern..

[37] Mikowicz M. (2016). Event-Based Control and Signal Processing.

[38] Socas R., Dormido S., Dormido R., Fabregas E. (2015). Event-based control strategy for mobile robots in wireless environment. Sensors.

[39] Xu W., Ho D.W.C., Li L., Cao J. (2017). Event-triggered schemes on leader-following consensus of general linear multiagent systems under different topologies. IEEE Trans. Cybern..

[40] Yi X., Lu W., Chen T. (2017). Pull-based distributed event-triggered consensus for multiagent systems with directed topologies. IEEE Trans. Neural Netw. Learn. Syst..

[41] Xie D., Xu S., Zhang B., Li Y., Chu Y. (2016). Consensus for multi-agent systems with distributed adaptive control and an event-triggered communication strategy. IET Control Theory Appl..

[42] Zhu W., Jiang Z.-P. (2015). Event-based leader-following consensus of multi-agent systems with input time delay. IEEE Trans. Autom. Control.

[43] Zhang X.-M., Han Q.-L., Zhang B.-L. (2017). An overview and deep investigation on sampled-data-based event-triggered control and filtering for networked systems. IEEE Trans. Ind. Inform..

[44] Dou C., Yue D., Guerrero J.M. (2017). Multiagent system-based event-triggered hybrid controls for high-security hybrid energy generation systems. IEEE Trans. Ind. Inform..

[45] Li H., Liao X., Huang T., Zhu W. (2015). Event-triggering sampling based leader-following consensus in second-order multi-agent systems. IEEE Trans. Autom. Control.

[46] Li H., Liu S., Soh Y.C., Xie L. (2017). Event-triggered communication and data rate constraint for distributed optimization of multiagent systems. IEEE Trans. Syst. Man Cybern. Syst..

[47] Acho L. (2017). Event-driven observer-based smart-sensors for output feedback control of linear systems. Sensors.

[48] Santos C., Martinez-Rey M., Espinosa F., Gardel A., Santiso E. (2017). Event-based sensing and control for remote robot guidance: An experimental case. Sensors.

[49] Heemels W.H., Donkers M.C.F., Teel A.R. (2013). Periodic event-triggered control for linear systems. IEEE Trans. Autom. Control.

[50] Choi J., Oh S., Horowitz R. (2009). Distributed learning and cooperative control for multi-agent systems. Automatica.

[51] Cortés J. (2009). Distributed kriged kalman filter for spatial estimation. IEEE Trans. Autom. Control.

[52] Lynch K.M., Schwartz I.B., Yang P. (2008). Decentralized environmental modeling by mobile sensor networks. IEEE Trans. Rob..

[53] Bhat S.P., Bernstein D.S. (2000). Finite-time stability of continuous autonomous systems. SIAM J. Control Optim..

